# Association between statin therapy and dementia risk: evidence from All of Us and UK Biobank with coenzyme Q7-related mediation analysis

**DOI:** 10.3389/fphar.2026.1915633

**Published:** 2026-10-29

**Authors:** Da Hoon Lee, Yoon-A. Park, Jung Sun Kim, Jeong Yee, Ji Min Han, Hye Sun Gwak

**Affiliations:** 1 College of Pharmacy and Graduate School of Pharmaceutical Sciences, Ewha Womans University, Seoul, Republic of Korea; 2 College of Pharmacy, Chungbuk National University, Cheongju, Republic of Korea; 3 Human-Centered Artificial Intelligence Research Institute, Ewha Womans University, Seoul, Republic of Korea; 4 Department of Pharmacy, Kangwon National University, Chuncheon, Republic of Korea; 5 School of Pharmacy, Sungkyunkwan University, Suwon, Republic of Korea

**Keywords:** COQ7, dementia, mediation analysis, pharmacoepidemiology, statins

## Abstract

**Background:**

Statins are widely prescribed for cardiovascular disease prevention in older adults; however, their association with dementia remains controversial. Given the high prevalence of both statin use and cognitive decline in aging populations, clarifying this relationship has critical implications for geriatric clinical practice and shared decision-making.

**Methods:**

We conducted a population-based retrospective cohort study using the All of Us Research Program (discovery cohort, n = 83,355; 31,513 statin users) and UK Biobank (replication cohort, n = 173,282; 43,976 statin users). Participants were adults aged ≥40 years who self-identified as White, followed from 2006 through 2023 (discovery) and 2017 (replication). Inverse probability of treatment weighting balanced baseline characteristics. Dementia risk was estimated using Cox proportional hazards models. An exploratory mediation analysis examined low-density lipoprotein cholesterol (LDL-C) and coenzyme Q7, a protein involved in CoQ10 biosynthesis, as potential pathways linking statin use and dementia.

**Results:**

Statin use was associated with significantly increased dementia risk in both cohorts (discovery: adjusted HR 2.22, 95% CI 2.02–2.44; replication: adjusted HR 2.54, 95% CI 1.98–3.33). Clear intensity-dependent relationships were observed, with high-intensity statins conferring 14% greater risk than moderate-to-low intensity therapy. In the exploratory mediation analysis, the estimated indirect effect through coenzyme Q7 was statistically significant and accounted for 8.2% of the total association, whereas the indirect effect through LDL-C was not statistically significant.

**Conclusion:**

Statin use was associated with higher dementia risk in both cohorts, and exploratory mediation findings suggested a modest statistical indirect association through COQ7, which should be considered hypothesis-generating. Given the observational design and conflicting evidence in the literature, these findings warrant further investigation before informing clinical decisions about statin therapy.

## 1 Introduction

Dementia represents one of the most critical public health challenges in geriatric medicine. As of 2019, an estimated 57 million individuals were living with dementia worldwide, projected to rise to 152 million by 2050 (Nichols et al., 2022). As aging populations expand globally, identifying modifiable risk factors for cognitive decline has become a critical priority in the absence of definitive therapeutic interventions.

Statins have become one of the most widely prescribed medication classes globally, with well-established effectiveness in reducing low-density lipoprotein cholesterol (LDL-C) levels and cardiovascular morbidity and mortality (Ridker et al., 2016; Collins et al., 2016). More than 25% of adults aged 40 and over in the United States reported statin use, with particularly high prevalence among the older population (Matyori et al., 2023). Given the substantial overlap between those prescribed statins and those at risk for cognitive decline, there is growing interest in understanding the potential relationship between statin use and dementia.

Current evidence remains inconclusive and contradictory. In 2012, the United States Food and Drug Administration updated statin labeling to include information about potential cognitive side effects, later clarifying in 2016 that such events were generally reversible (U.S. Food and Drug Administration, 2016). Some observational studies have suggested that statin use may increase dementia risk (Roy et al., 2020; Ye et al., 2025), while others have found no association (Ott et al., 2015; Zhou et al., 2021), and recent meta-analysis reported even protective effects (Westphal Filho et al., 2025).

The controversial nature of this relationship has led to diverse mechanistic explanations. Potential neuroprotective pathways include anti-inflammatory effects and cardiovascular benefits that may reduce cerebrovascular disease burden (Liao and Laufs, 2005; Law et al., 2003). Conversely, harmful mechanisms have been suggested, including cerebral cholesterol depletion and mitochondrial dysfunction through depletion of coenzyme Q10 (CoQ10) (Orth and Bellosta, 2012; Fernández-Portero et al., 2023), a compound reduced by statin therapy (Qu et al., 2018). Understanding the role of CoQ10 and LDL-C may offer insights into the biological mechanisms underlying statin-associated cognitive effects.

Because direct measurements of circulating CoQ10 are not currently available in large-scale proteomic resources such as the UK Biobank, alternative biomarkers reflecting CoQ10 biosynthesis are needed. Coenzyme Q7 (COQ7) is a key enzyme involved in the late stages of CoQ10 biosynthesis and has been proposed as a representative marker of CoQ10 biosynthetic capacity. In our recent study investigating statin-associated muscle symptoms (Lee et al., 2026), we successfully utilized plasma COQ7 levels as a proxy for CoQ10 biosynthesis and demonstrated that impaired COQ7-related pathways were associated with statin-induced toxicity. Therefore, we hypothesized that COQ7 may also help generate hypotheses about the biological pathways underlying the relationship between statin therapy and dementia.

To address these knowledge gaps, we conducted a comprehensive population-based study using two large, independent cohorts—the All of Us Research Program and the UK Biobank—focusing on participants aged 40 years or older who self-identified as White. Our study aimed to evaluate the epidemiological association between statin use and incident dementia, and explore potential biological mechanisms.

## 2 Materials and methods

### 2.1 Study design and setting

We conducted a population-based retrospective cohort study utilizing two large-scale biomedical resources: the All of Us Research Program and the UK Biobank. To explore potential biological mechanisms, we incorporated proteomic data from the UK Biobank Pharma Proteomics Project (UKB-PPP). All research procedures adhered to the Declaration of Helsinki. Written or electronic informed consent was obtained from all participants.

### 2.2 Discovery cohort from the All of Us Research Program

The All of Us Research Program is a large-scale, prospective, longitudinal cohort study launched by the U.S. National Institutes of Health in 2018, aiming to enroll at least one million adults aged 18 years and older from across the United States (The All of Us Research Program Investigators, 2019). As of 2025, the program has enrolled approximately 600,000 individuals. Data collected include health questionnaires, electronic health records (EHRs), physical measurements, digital health technology, and genomic data. For this study, we accessed the All of Us Controlled Tier Dataset v8 (C2024Q3R4; released February 2025), with follow-up data available through 1 October 2023. The protocol was reviewed by the Institutional Review Board of the All of Us Research Program, following the regulations and guidance of the National Institutes of Health Office for Human Research Protections.

Statin exposure was defined using prescription records from 1 January 2006 to 1 October 2022, and participants with any statin prescription on or before 31 December 2005 were excluded to restrict the analysis to new users. Statin users were those with a first statin prescription during this period, which served as the index date. Non-users were those with no statin prescription throughout the observation period; this definition therefore relied on information after the index date and may be susceptible to time-related selection bias. Each non-user was assigned a pseudo-index date randomly sampled from the distribution of index dates among users, restricted to the period during which the participant was alive and under observation. In both groups, participants with dementia before the (pseudo-)index date were excluded, and follow-up started at the (pseudo-)index date.

Statin medications were identified using the Anatomical Therapeutic Chemical classification code C10AA (HMG-CoA reductase inhibitors), including atorvastatin, fluvastatin, lovastatin, pitavastatin, pravastatin, rosuvastatin, and simvastatin. Statins were classified by clinical intensity and lipophilicity as detailed in Supplementary Methods.

Eligible participants were adults aged ≥40 years with available EHR and *APOE* genotype data who self-identified as White ethnicity. We excluded individuals with a recorded statin prescription before 1 January 2006 (to identify participants whose first recorded statin prescription occurred during the study period); those whose first recorded statin prescription occurred after 1 October 2022 (to ensure sufficient time for outcome ascertainment); participants with prevalent dementia or neurological conditions before the index date (stroke, Parkinson’s disease, or traumatic brain injury); and those with observation duration <1 year (to ensure adequate exposure duration and outcome assessment) (Evans and Golomb, 2009).

The outcome was incident dementia, defined as the first occurrence of any dementia diagnosis during follow-up. Dementia cases were identified using standardized coding systems: International Classification of Diseases, Ninth Revision (ICD-9), International Classification of Diseases, Tenth Revision (ICD-10), and Systematized Nomenclature of Medicine (SNOMED) codes (Supplementary Table S1). For each participant, the observation period began at the index date and ended at the time of censoring, defined as the earliest occurrence of incident dementia, discontinuation of statin therapy among users, loss to follow-up, death, or study completion (1 October 2023).

### 2.3 Replication cohort from the UK Biobank

The UK Biobank represents a large-scale prospective population-based study that enrolled approximately 500,000 participants aged 40–69 years across England, Wales, and Scotland during 2006-2010 (Bycroft et al., 2018). The study collected data from questionnaires, interviews, EHRs, genotype data, and protein level data. Follow-up data are available through 31 May 2022 for inpatient records, and 31 May 2017 for primary records. Ethical approval was obtained from the National Research Ethics Committee (16/NW/0274) under application ID 126305.

Eligible participants met the same inclusion and exclusion criteria as the discovery cohort, except only those with first recorded statin prescription between 1 January 2006 and 31 May 2016 were included. White ethnicity was defined as British, Irish, or other White background. Statin exposure and index date definitions were identical to the discovery cohort. Statin medications were identified using the read code and drug name (Supplementary Table S2). Dementia cases were identified using ICD-9, ICD-10, and Read codes (Supplementary Table S1). The observation period extended from index to dementia diagnosis, statin discontinuation among users, death, loss to follow-up, or 31 May 2017.

### 2.4 Mediation analysis cohort from UKB-PPP

To explore potential biological pathways linking statin use to dementia, we conducted a mediation analysis using proteomic data from the UKB-PPP. Because direct CoQ10 measurements were unavailable, we evaluated COQ7, a protein involved in CoQ10 biosynthesis, as a potential mediator (Acosta et al., 2016). We constructed a cohort from UKB-PPP participants based on self-reported medication use at enrollment. Participants were classified as statin users or non-users based on reported regular statin use. Statin medications were coded using standardized classification systems (Supplementary Table S3). Dementia case definitions were identical to those used in the replication cohort. To examine LDL-C as a second mediator, we excluded participants with missing LDL-C measurements. Detailed methodology is provided in Supplementary Methods.

### 2.5 Associations of COQ7 and LDL-C with dementia

To complement the mediation analyses, we examined the associations of COQ7 and LDL-C levels with incident dementia in the UKB-PPP cohort. These analyses used the same cohort definitions, baseline statin exposure, and dementia outcome as the corresponding mediation analyses. Each biomarker was evaluated separately among participants with available measurements to assess its association with dementia and characterize the exposure–response relationship.

### 2.6 Covariates

Covariates included demographic characteristics, genetic factors, comorbidities, and concomitant medications. Age and index year were defined at the index date. BMI was based on the measurement temporally closest to the index date. Comorbidities were ascertained using all available diagnostic records recorded on or before the index date, and concomitant medication use was defined based on prescriptions recorded during the 365 days preceding the index date. Sex and *APOE* genotype were treated as time-invariant. Detailed definitions and coding algorithms are provided in the Supplementary Methods.

### 2.7 Statistical analysis

Baseline characteristics were compared between statin users and non-users using standardized mean differences (SMDs), with values <0.1 indicating negligible imbalance. To account for baseline differences between statin users and non-users, we implemented inverse probability of treatment weighting (IPTW) across all cohorts using propensity scores derived from multivariable logistic regression models. To reduce the influence of extreme weights, we truncated weights at the 99.5th percentile. Unless otherwise specified, analyses were performed using IPTW weights.

Time-to-event analysis employed Cox proportional hazards regression to estimate hazard ratios (HRs) with 95% confidence intervals (CI) and adjusted HRs (aHRs) after adjusting for all covariates. The proportional hazards assumption was verified using Schoenfeld residuals. Kaplan-Meier survival curves illustrated cumulative dementia incidence by statin exposure status. We conducted further analyses examining the associations of statin intensity and lipophilicity on dementia risk. Direct comparisons were performed within each stratum to assess intensity-dependent relationships and differential effects by statin type. As an additional analysis, we conducted stratified analyses across pre-specified demographic and clinical strata including sex, age groups, body mass index (BMI) categories, *APOE* status, comorbidities, and medications.

We performed an exploratory mediation analysis to investigate whether COQ7 or LDL-C statistically accounted for part of the association between statin use and dementia risk. Each biomarker was evaluated separately within a counterfactual-based mediation framework (Gentreau et al., 2023; VanderWeele, 2016). Because statin use and biomarker levels were assessed concurrently, these analyses were considered hypothesis-generating. COQ7 and LDL-C were rank-inverse normal transformed, with effect estimates presented per standard deviation (SD) change. Two regression models were constructed for each mediator: a mediator model (β_1_), and an outcome model (β_2_). The direct effect, indirect effect, total effect, and proportion mediated were estimated using nonparametric bootstrapping with 1,000 simulations (R *mediation* package) (Tingley et al., 2014). The proportion mediated was calculated as the ratio of the indirect effect to the total effect (VanderWeele, 2015). We additionally examined differences in COQ7 levels between users of individual statins and non-users, as well as among statin types.

Associations of COQ7 and LDL-C levels with incident dementia were assessed using logistic regression, with statin use included as a covariate. Biomarker levels were modeled as continuous linear terms and using restricted cubic splines with three knots at the 10th, 50th, and 90th percentiles. In the linear models, odds ratios (ORs) were estimated per one-unit increase in the transformed biomarker level. In the spline models, the median biomarker level was used as the reference. Overall associations were evaluated by jointly testing the linear and nonlinear spline terms, and nonlinearity was assessed by testing the nonlinear term. Both tests used robust Wald statistics. Sandwich standard errors were used to calculate 95% CIs. Details of statistical software and procedures are described in Supplementary Methods.

### 2.8 Sensitivity analyses

We conducted three sensitivity analyses to evaluate the robustness of the primary findings. First, we repeated the IPTW-weighted Cox analyses after restricting both cohorts to participants with at least 3 years of observed follow-up after the index date. The propensity scores and IPTW weights from the primary analyses were retained, and the adjusted models included the same baseline covariates as the primary outcome models.

Second, we applied propensity-score overlap weighting as an alternative weighting approach in both cohorts. This method emphasizes participants with comparable probabilities of statin use based on measured baseline characteristics while reducing the influence of those with extreme propensity scores. Propensity scores were estimated using the same baseline covariates as in the primary analyses. Covariate balance was evaluated using SMD. Overlap-weighted Cox proportional hazards models with robust standard errors were used to estimate HRs and 95% CIs. The adjusted models additionally included the same baseline covariates as the primary outcome models.

Third, to account for competing mortality, we fitted Fine–Gray subdistribution hazards models in both cohorts, treating death before dementia as a competing event. The models incorporated the same IPTW and covariate adjustment as the primary Cox analyses. Results are reported as adjusted subdistribution hazard ratios (asHRs) with 95% CIs.

## 3 Results

### 3.1 Discovery cohort

In the discovery cohort, the final cohort comprised 83,355 participants (31,513 statin users) (Figure 1A). The mean observation period was 7.9 ± 4.7 years for statin users and 8.6 ± 4.6 years for non-users. Before IPTW, substantial baseline imbalances were evident. Statin users were more frequently male (52.0% vs. 38.2%, SMD = 0.280), older (≥70 years: 24.9% vs. 21.0%), and had higher BMI (>30 kg/m^2^: 43.5% vs. 31.7%, SMD = 0.337). Comorbidity burden was markedly higher among statin users, including hypertension (49.5% vs. 16.1%, SMD = 0.760), diabetes mellitus (18.4% vs. 3.7%, SMD = 0.481), and coronary artery disease (17.5% vs. 3.6%, SMD = 0.466). IPTW successfully balanced baseline characteristics (all SMDs <0.1), yielding weighted cohorts of 81,665.2 users and 83,440.5 non-users (Figure 1B; Supplementary Table S4).

**FIGURE 1:**
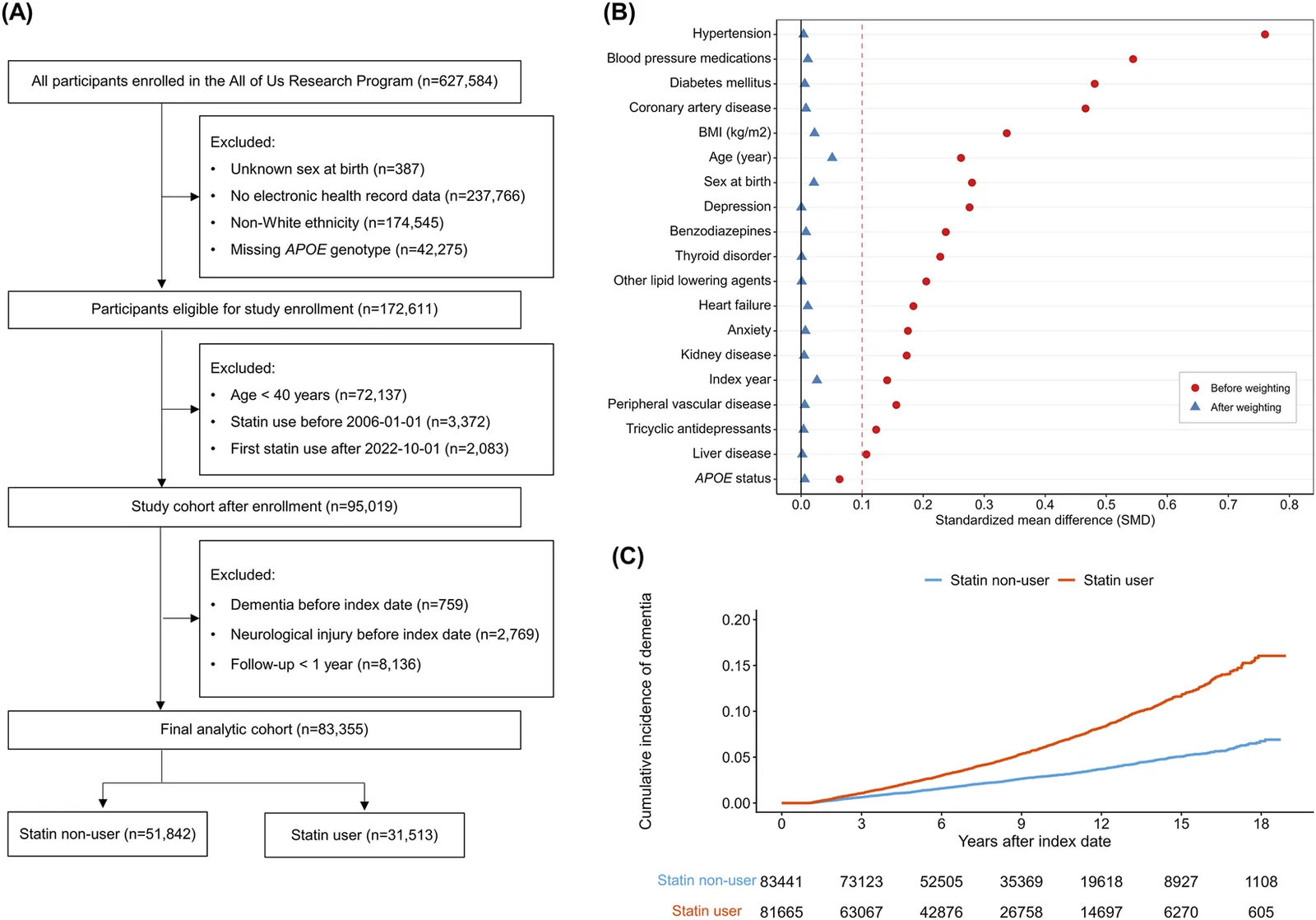
Participant selection, covariate balance, and cumulative dementia incidence in the discovery cohort. **(A)** Flowchart depicting participant selection from the All of Us Research Program. **(B)** SMDs for baseline covariates before and after IPTW. SMD values below 0.1 (indicated by the vertical dashed line) represent negligible imbalance between statin users and non-users. After IPTW, all covariates achieved balance with SMD <0.1. Age was categorized into four groups: <50, 50–59, 60–69, and ≥70. BMI was grouped into three categories: <25, 25–30, and >30. **(C)** Kaplan-Meier survival curves illustrating cumulative dementia incidence by statin exposure status. Abbreviations: APOE, apolipoprotein E; BMI, body mass index; IPTW, inverse probability of treatment weighting; SMD, standardized mean difference.

A total of 5,968 incident dementia cases were identified. Kaplan-Meier analysis demonstrated progressively diverging cumulative incidence, with statin users experiencing consistently higher dementia rates (Figure 1C). Statin use was associated with significantly increased dementia risk (aHR: 2.22, 95% CI: 2.02–2.44, *P* < 0.001), with incidence rates of 64.9 versus 30.7 per 10,000 person-years in users versus non-users (Table 1). Analyses by dementia subtype showed consistent associations: Alzheimer’s disease (aHR: 2.02, 95% CI: 1.18–3.45), vascular dementia (aHR: 2.30, 95% CI: 1.38–3.79), and other dementia types (aHR: 2.23, 95% CI: 2.02–2.46).

**TABLE 1:** Risk of all-cause and subtype dementia by statin use in the discovery cohort.

Dementia and subtypes	Case	Control	Person-years	Incidence rate^†^	HR (95% CI)	
					Unadjusted	Adjusted^‡^
All types of dementia						
Statin non-user	2,148	81,292	699,981.3	30.7	1 (ref)	1 (ref)
Statin user	3,820	77,846	588,638.4	64.9	2.20 (2.01–2.41)^***^	2.22 (2.02–2.44)^***^
Alzheimer’s disease						
Statin non-user	86	81,292	686,415.7	1.2	1 (ref)	1 (ref)
Statin user	131	77,846	563,008.4	2.3	1.98 (1.22–3.24)^**^	2.02 (1.18–3.45)^*^
Vascular dementia						
Statin non-user	76	81,292	686,287.5	1.1	1 (ref)	1 (ref)
Statin user	133	77,846	562,834.9	2.4	2.22 (1.35–3.65)^**^	2.30 (1.38–3.79)^**^
Other dementias						
Statin non-user	1,986	81,292	698,910.1	28.4	1 (ref)	1 (ref)
Statin user	3,555	77,846	586,661.0	60.2	2.22 (2.02–2.44)^***^	2.23 (2.02–2.46)^***^

Data are derived from weighted proportions and are not absolute numbers.

Abbreviations: CI, confidence interval; HR, hazard ratio.

† Incidence rate per 10,000 person-years.

Adjusted for sex, age groups, index year, BMI categories, APOE status, comorbidities, and medications.

*P < 0.05, **P < 0.01, ***P < 0.001.

Analysis by statin intensity revealed clear intensity-dependent relationships (Table 2). After excluding 2,695 users with missing intensity data, Model 1 showed that moderate-to-low intensity use was associated with elevated dementia risk versus non-use (aHR: 2.08, 95% CI: 1.87–2.30, *P* < 0.001; incidence rate 59.0 per 10,000 person-years), and high-intensity use showed even greater risk (aHR: 2.70, 95% CI: 2.38–3.06, *P* < 0.001; incidence rate 84.6 per 10,000 person-years). In Model 2, high-intensity use showed higher dementia risk versus moderate-to-low intensity (aHR: 1.14, 95% CI: 1.07–1.21, *P* < 0.001).

**TABLE 2:** Dementia risk by statin intensity in the discovery cohort.

​	Case	Control	Person- years	Incidence rate^†^	Model 1		Model 2	
					Unadjusted HR (95% CI)	Adjusted HR^‡^ (95% CI)	Unadjusted HR (95% CI)	Adjusted HR^‡^ (95% CI)
Statin non-users	2,148	81,292	699,981.3	30.7	1 (ref)	1 (ref)	-	-
Statin user §								
Moderate-to-low intensity	2,221	48,315	376,210.0	59.0	2.00 (1.79–2.20)^***^	2.08 (1.87–2.30)^***^	1 (ref)	1 (ref)
High intensity	1,328	22,245	157,033.2	84.6	2.95 (2.62–3.32)^***^	2.70 (2.38–3.06)^***^	1.22 (1.15–1.29)^**^	1.14 (1.07–1.21)^***^

Data are derived from weighted proportions and are not absolute numbers. Model 1 compares each statin intensity group to statin non-users; Model 2 compares high-intensity to moderate-to-low intensity use among statin users.

Abbreviations: CI, confidence interval; HR, hazard ratio.

† Incidence rate per 10,000 person-years.

‡ Adjusted for sex, age groups, index year, BMI categories, APOE status, comorbidities, and medications.

§ Data not available for all patients.

*P < 0.05, **P < 0.01, ***P < 0.001.

Both lipophilic and hydrophilic statins were associated with increased dementia risk (Supplementary Table S5). Lipophilic statins showed aHR 2.14 (95% CI: 1.94–2.36, *P* < 0.001) with incidence rate 62.5 per 10,000 person-years, and hydrophilic statins had aHR 2.50 (95% CI: 2.18–2.87, *P* < 0.001) with incidence rate 73.6 per 10,000 person-years. Within each intensity category, hydrophilic statins showed slightly higher dementia risk than lipophilic statins in both high-intensity (aHR: 1.10; 95% CI: 0.88–1.37) and moderate-to-low intensity groups (aHR: 1.15; 95% CI: 0.97–1.37), though neither difference reached statistical significance (Supplementary Table S6).

Stratified analysis revealed consistent associations across demographic and clinical strata (Supplementary Figure S1). The association between statin use and higher dementia risk was observed across all age groups examined. As *APOE* ε4 is a well-established genetic risk factor for cognitive decline (Caselli et al., 2004), the association between statin use and dementia persisted across all *APOE* ε4 strata. Among participants with peripheral vascular disease or those using other lipid-lowering medications, aHRs were elevated but not statistically significant, likely reflecting limited statistical power due to smaller subgroup sizes (Serdar et al., 2021).

### 3.2 Replication analysis

In the replication cohort, the final cohort comprised 173,282 participants (43,976 statin users) (Figure 2A). The mean observation period was 6.1 ± 3.1 years for statin users and 7.2 ± 3.4 years for non-users. Pre-weighting imbalances paralleled those observed in the discovery cohort. IPTW successfully balanced baseline characteristics, yielding weighted cohorts of 167,265.5 users and 176,103.9 non-users (Figure 2B; Supplementary Table S7).

**FIGURE 2:**
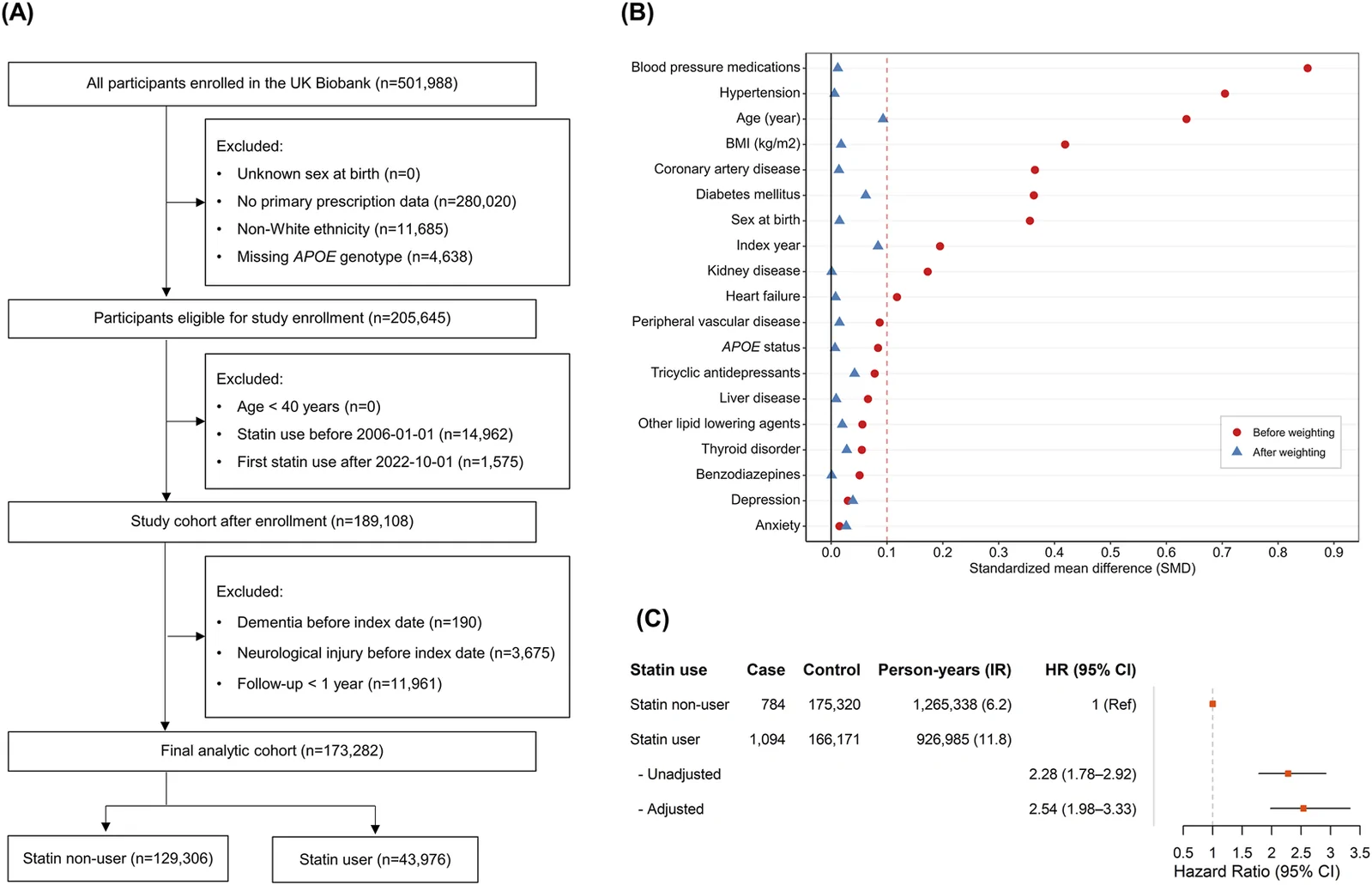
Participant selection, covariate balance, and replication analysis in the replication cohort. **(A)** Flowchart depicting participant selection from the UK Biobank. **(B)** SMDs for baseline covariates before and after IPTW. SMD values below 0.1 (indicated by the vertical dashed line) represent negligible imbalance between statin users and non-users. After IPTW, all covariates achieved balance with SMD <0.1. Age was categorized into four groups: <50, 50–59, 60–69, and ≥70. BMI was grouped into three categories: <25, 25–30, and >30. **(C)** Forest plot displaying the analysis results comparing dementia risk between statin users and non-users. Adjusted HRs were adjusted for sex, age groups, index year, BMI categories, *APOE* status, comorbidities, and medications. Incidence rates are expressed as per 10,000 person-years. Abbreviations: APOE, apolipoprotein E; BMI, body mass index; HR, hazard ratio; IPTW, inverse probability of treatment weighting; IR, incidence rate; SMD, standardized mean difference.

The previously observed association between statin use and dementia risk was replicated (Figure 2C). The incidence rate of dementia was 11.8 per 10,000 person-years among statin users, compared to 6.2 per 10,000 person-years in non-users. Statin use was associated with a significantly higher dementia risk, with an aHR of 2.54 (95% CI: 1.98–3.33; *P* < 0.001).

### 3.3 Sensitivity analyses

The associations were consistent across all three sensitivity analyses in both cohorts (all *P* < 0.001). When the analyses were restricted to participants with at least 3 years of observed follow-up, statin use remained associated with a higher dementia hazard in the discovery cohort (aHR: 2.27; 95% CI: 2.04–2.54) and the replication cohort (aHR: 2.72; 95% CI: 2.13–3.40) (Supplementary Table S8). Overlap weighting balanced all measured baseline covariates (all SMDs <0.1) and yielded similar estimates (discovery cohort: aHR, 2.18; 95% CI, 2.00–2.38; replication cohort: aHR, 2.75; 95% CI, 2.30–3.29) (Supplementary Table S9). In Fine–Gray models treating death as a competing event, the associations also persisted (discovery cohort: asHR, 2.19; 95% CI, 2.00–2.40; *P* < 0.001; replication cohort: asHR, 2.49; 95% CI, 2.04–3.08; *P* < 0.001).

### 3.4 Mediation analysis

IPTW achieved covariate balance across groups in the final analytic cohort of 38,553 participants (6,108 statin users) (Supplementary Table S10). For LDL-C mediation analysis, 36,587 participants were included after excluding those with missing LDL-C data. Statin users had significantly lower COQ7 (0.11 SD decrease) and LDL-C levels (0.82 SD decrease) compared to non-users (both *P* < 0.001) (Supplementary Table S11). Atorvastatin and simvastatin users had significantly lower COQ7 levels than non-users, and no pairwise differences among statin types remained statistically significant after Holm correction (Supplementary Tables S12 and S13).

The conceptual framework for the mediation analyses is illustrated in Figure 3A. The estimated indirect association through COQ7 was statistically significant, whereas that through LDL-C was not (Figure 3B). Statin use was associated with lower COQ7 levels (β_1_: −0.10, 95% CI: −0.12 to −0.09), and lower COQ7 was associated with higher odds of dementia (β_2_: −0.10, 95% CI: −0.18 to −0.03), yielding a statistically significant estimated indirect effect (β: 0.0005, 95% CI: 0.0001–0.0010), that accounted for 8.2% of the total association.

**FIGURE 3:**
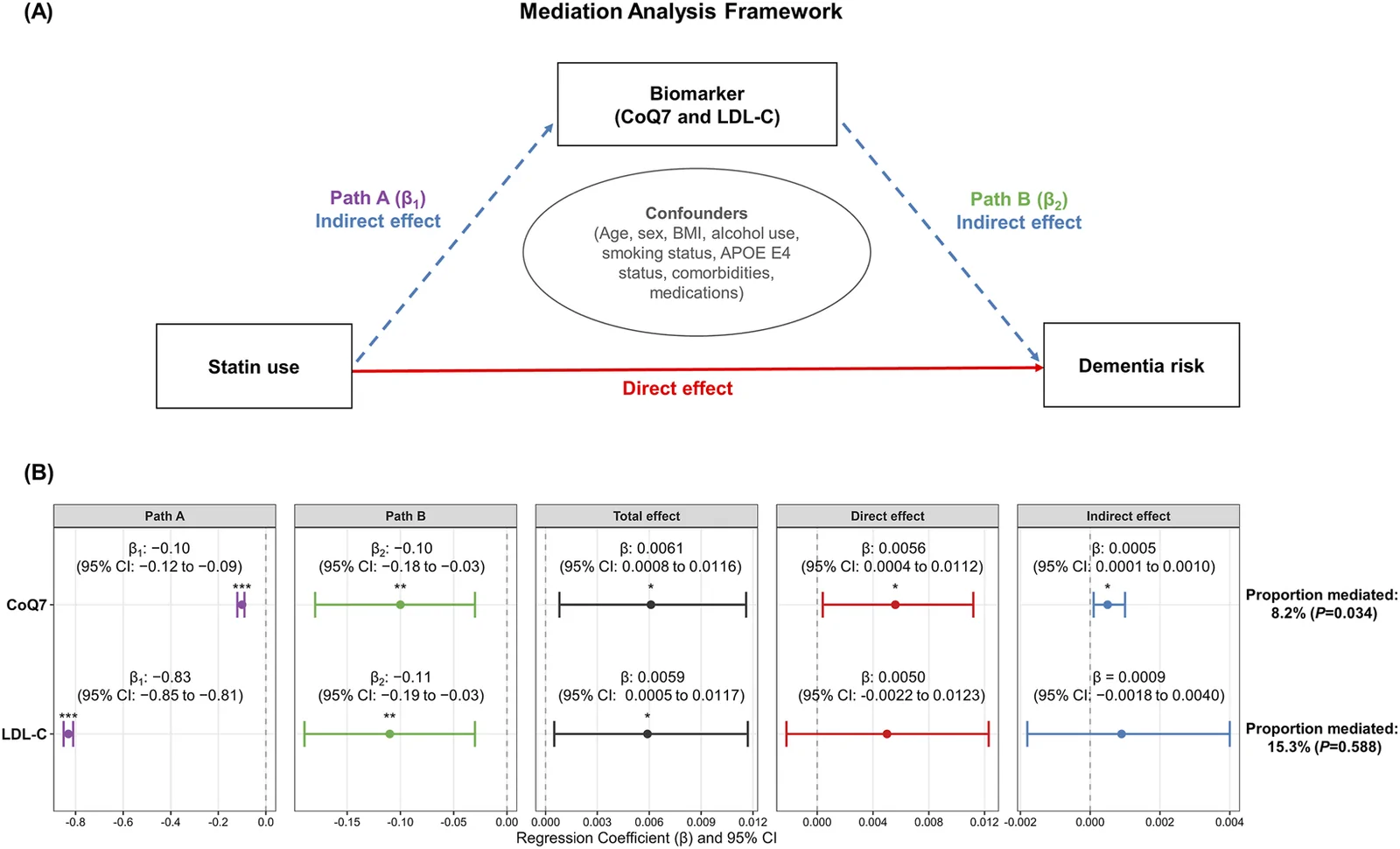
Exploratory mediation analysis examining the role of COQ7 and LDL-C in the association between statin use and dementia risk. **(A)** Conceptual framework illustrating the mediation analysis model. Two mediators were examined independently: COQ7 and LDL-C. For each mediator, two regression models were constructed: a mediator model estimating the association of statin use with the biomarker level (Path A, β1), and an outcome model assessing the effect of the biomarker on dementia risk (Path B, β2). The indirect effect is represented by dotted arrows, while the direct effect is shown by continuous arrows. **(B)** Results of the mediation analyses. The analysis included 38,553 participants from the UK Biobank Pharma Proteomics Project; for LDL-C mediation analysis, 36,587 participants were included after excluding those with missing LDL-C data. Proportions mediated and P values for the indirect effects are shown for each mediator. Abbreviations: β, regression coefficient; CI, confidence interval; COQ7, coenzyme Q7; CoQ10, coenzyme Q10; LDL-C, low-density lipoprotein cholesterol. ^*^ *P* < 0.05, ^**^ *P* < 0.01, ^***^ *P* < 0.001.

In contrast, although statin use was strongly associated with reduced LDL-C (β_1_: −0.83, 95% CI: −0.85 to −0.81), and lower LDL-C was also associated with higher odds of dementia (β_2_: −0.11, 95% CI: −0.19 to −0.03), the estimated indirect effect through LDL-C was not statistically significant (β: 0.0009, 95% CI: −0.0018–0.0040). Detailed results of these analyses are provided in Supplementary Table S14.

### 3.5 Associations of COQ7 and LDL-C with dementia

Higher COQ7 levels were significantly associated with lower odds of dementia (OR: 0.851, 95% CI: 0.744–0.973; *P* = 0.018), whereas LDL-C levels were not significantly associated with dementia (OR: 0.955, 95% CI: 0.832–1.095; *P* = 0.509). Restricted cubic spline analysis showed an overall association between COQ7 levels and dementia (*P* = 0.009), with evidence of nonlinearity (*P* = 0.044). Lower COQ7 levels were associated with higher odds of dementia relative to the median, with a flattening of the curve above the median. No significant overall association or nonlinearity was observed for LDL-C (*P* for overall association = 0.700; *P* for nonlinearity = 0.887) (Supplementary Figure S2).

## 4 Discussion

Our study revealed an association between statin use and dementia risk following adjustment for baseline confounders through IPTW. Statin users exhibited significantly higher rates of incident dementia, including Alzheimer’s disease and vascular dementia, compared to statin non-users. The primary findings were consistent across three sensitivity analyses in both cohorts. Also, high-intensity statin use was associated with a higher dementia hazard than moderate-to-low-intensity use. This elevated risk remained consistent across diverse demographic and clinical subpopulations.

Exploratory mediation analyses further showed a statistically significant estimated indirect association through COQ7, a protein involved in CoQ10 biosynthesis, suggesting a potential pathway for further investigation. The additional biomarker–dementia analyses showed that lower COQ7 levels were associated with higher odds of dementia. The spline analysis suggested that the association may be more pronounced at lower COQ7 levels rather than uniform across the observed range. These exploratory findings are compatible with a role for CoQ10 biosynthesis-related processes in the observed association.

Our findings align with prior studies exploring associations between statin use and cognitive outcomes. Previous UK Biobank research found associations between statin use and increased Alzheimer’s disease risk, although suggested potential protective effects among *APOE* ε4 homozygotes (Ye et al., 2025). In contrast, our study demonstrated consistent increases in dementia risk across all *APOE* ε4 genotypes, indicating that cognitive effects of statins may be independent of genetic susceptibility. Another UK Biobank study found that statin use was associated with increased white matter hyperintensity volumes and reduced gray matter volumes after nine years—structural changes indicative of neurodegeneration and accelerated brain aging (Gentreau et al., 2024). Consistent with our results, other research has demonstrated intensity-dependent relationships in cognitive dysfunction, with high-intensity users showing significantly higher rates of cognitive impairment compared to moderate-intensity therapy (Roy et al., 2020).

We observed substantial differences in dementia incidence rates between the discovery and replication cohorts. The discovery cohort showed higher rates (64.9 vs. 30.7 per 10,000 person-years for statin users vs. non-users) compared to the replication cohort (11.8 vs. 6.2 per 10,000 person-years). This discrepancy may be partly attributable to differences in data availability, as the discovery cohort was followed through October 2023 while the replication cohort was limited to May 2017. When we restricted the discovery cohort analysis to a comparable follow-up period ending in May 2017, incidence rates became more similar between cohorts (16.9 vs. 3.4 and 15.0 vs. 3.9 per 10,000 person-years, respectively), suggesting that differential follow-up time substantially contributed to the observed discrepancy.

Our exploratory mediation findings point to COQ7 as a candidate biomarker that may warrant further investigation. Although no prior studies have examined the effect of statins on COQ7, previous studies have consistently demonstrated statin-induced reductions in CoQ10 concentrations (Qu et al., 2018). Because COQ7 plays an essential role in CoQ10 biosynthesis (Acosta et al., 2016), lower COQ7 levels could reflect reduced CoQ10 biosynthetic capacity, which has been linked to mitochondrial dysfunction and neuronal damage (Desbats et al., 2015; Wang et al., 2022).

The use of COQ7 as a biosynthesis-related biomarker is consistent with our recent pharmacogenomic study of statin-associated muscle symptoms (SAMS), in which COQ7 protein levels were used as a proxy for CoQ10 biosynthesis because direct CoQ10 measurements were unavailable in large-scale proteomic datasets (Lee et al., 2026). In that study, genetic variants associated with lower COQ7 levels were associated with an increased risk of SAMS, suggesting that CoQ10 biosynthesis-related processes may be relevant to statin-associated adverse effects. Whether a similar pathway contributes to cognitive outcomes remains to be determined in longitudinal and experimental studies.

CoQ10 serves as a critical antioxidant and mitochondrial electron carrier that protects neurons from oxidative damage and maintains cellular energy production in the brain (Littarru and Tiano, 2007; Kure et al., 2016). Therefore, reductions in CoQ10 biosynthetic capacity may increase neuronal vulnerability and accelerate neurodegenerative processes. Consistent with this hypothesis, population-based studies have reported that CoQ10 supplementation is associated with a lower risk of cognitive decline (Momiyama, 2014).

Importantly, LDL-C was not significantly associated with dementia in either the linear or spline analyses, and its indirect effect in the mediation analysis was not statistically significant. Thus, the observed association was not explained by LDL-C reduction in these analyses. Previous studies evaluating the role of LDL-C in dementia have reported inconsistent findings (Gentreau et al., 2023; Guo et al., 2020; Jurcau and Simion, 2021; Rej et al., 2016), and a recent long-term randomized controlled trial demonstrated no evidence of cognitive harm despite sustained exposure to very low LDL-C levels (Zimerman et al., 2025).

Taken together, these findings suggest a possible contribution of CoQ10 biosynthesis-related processes to the observed statin–dementia association, although they do not exclude a role for cholesterol-related pathways. However, the estimated indirect association through COQ7 accounted for only 8.2% of the total association and should be regarded as hypothesis-generating. The clinical implications require further investigation, as the proportion mediated alone does not quantify the absolute impact on dementia risk.

This study has limitations inherent to its observational design, which restricts causal inference despite robust adjustment strategies. Although IPTW improved balance in measured baseline covariates, residual confounding and confounding by indication may remain, as underlying cardiovascular risk may influence both statin prescribing and dementia risk. In particular, smoking and alcohol use were excluded from the prescription-based analyses because they were assessed only at enrollment and might not reflect participants’ status at the index date, leaving potential residual confounding by these factors. Additional limitations concern the mediation analysis. Direct CoQ10 measurements were unavailable, and COQ7 protein levels were used as a biosynthesis-related biomarker rather than a direct measure of CoQ10. Because COQ7 was measured only once during regular statin use and pre-treatment measurements were unavailable, the temporal ordering between statin exposure and COQ7 levels could not be established. The mediation models also relied on the unverifiable assumption of no unmeasured confounding and assumed a linear biomarker–dementia association, which may not hold for COQ7 given its possible nonlinear association. Accordingly, the mediation findings should be interpreted as exploratory statistical associations rather than evidence of a causal mechanism, and require external validation because COQ7 measurements were available only in UKB-PPP. Finally, baseline self-reported statin use did not provide the dose information needed to evaluate intensity-related differences in COQ7 levels.

This study has notable strengths that enhance the robustness of its findings. First, we leveraged two large-scale population cohorts—All of Us Research Program and the UK Biobank—providing substantial statistical power to detect associations in low-incidence outcomes. The use of independent cohorts with differing population characteristics allowed cross-validation of results, thereby strengthening external validity. Second, we employed IPTW to adjust for confounding while preserving the full analytic sample, which is particularly effective for rare outcomes (Yoshida et al., 2017). Finally, integrating biomarker-based mediation analyses provided complementary evidence to prioritize COQ7-related biological hypotheses for further investigation.

## 5 Conclusion

In conclusion, statin use was associated with higher dementia risk in two independent cohorts, and high-intensity use was associated with a higher dementia hazard than moderate-to-low-intensity use. Exploratory mediation analyses suggested a modest statistical indirect association through COQ7, which warrants confirmation in longitudinal and experimental studies. Given the observational design and conflicting evidence in the literature, further studies are needed to clarify the observed associations and their clinical implications.

## Data Availability

The data analyzed in this study is subject to the following licenses/restrictions: The datasets analyzed during the current study are available in the All of Us Research Program and the UK Biobank. Restrictions apply to the availability of these data, which were used under license for the current study. Access to these data can be obtained by registered researchers through the respective institutional application procedures. Requests to access these datasets should be directed to All of Us Research Program repository (https://www.researchallofus.org/) and the UK Biobank repository (https://www.ukbiobank.ac.uk/).
