# Supplementary material for: Association between statin therapy and dementia risk: evidence from All of Us and UK Biobank with coenzyme Q7-related mediation analysis
Source: Front Pharmacol. 2026 Oct 29;17:1915633. doi: 10.3389/fphar.2026.1915633 (PMC13755691; doi:10.3389/fphar.2026.1915633)
Supplement: Supplementary file 1 [file Supplementaryfile1.docx]

**Association between statin therapy and dementia risk: evidence from All of Us and UK Biobank with coenzyme Q7-related mediation analysis**

**Supplementary Data**

- Supplementary Methods
- Supplementary Table 1. Diagnostic codes used to define all-cause dementia and dementia subtypes
- Supplementary Table 2. Read codes and drug names used to identify statin prescriptions in the UK Biobank
- Supplementary Table 3. Medication codes used to identify statin use in the UK Biobank based on baseline survey responses
- Supplementary Table 4. Baseline characteristics of statin users and non-users before and after inverse probability of treatment weighting in the discovery cohort.
- Supplementary Table 5. Association between statin type and dementia risk in the discovery cohort.
- Supplementary Table 6. Comparison of dementia risk by statin type within statin intensity groups in the discovery cohort.
- Supplementary Table 7. Baseline characteristics of statin users and non-users before and after inverse probability of treatment weighting in the replication cohort.
- Supplementary Table 8. Association between statin use and incident dementia among participants with at least 3 years of observed follow-up.
- Supplementary Table 9. Overlap-weighted sensitivity analyses of the association between statin use and incident dementia
- Supplementary Table 10. Baseline characteristics of statin users and non-users before and after inverse probability of treatment weighting in the mediation analysis cohort.
- Supplementary Table 11. Distribution of COQ7 and LDL-C levels in statin users and non-users.
- Supplementary Table 12. Adjusted differences in baseline COQ7 levels between users of individual statins and non-users in the UKB-PPP cohort.
- Supplementary Table 13. Pairwise comparisons of baseline COQ7 levels among statin users in the UKB-PPP cohort.
- Supplementary Table 14. Mediation analyses of the association between statin use and dementia risk with COQ7 and LDL-C.
- Supplementary Figure 1. Stratified analysis of the association between statin use and dementia risk in the discovery cohort.
- Supplementary Figure 2. Associations of COQ7 and LDL-C levels with incident dementia in the UKB-PPP cohort.

**Supplementary Methods**

**Statin classification methodology**

Statins were categorized by clinical intensity following established guidelines: high-intensity (atorvastatin 40–80 mg and rosuvastatin 20–40 mg), moderate-intensity (atorvastatin 10–20 mg, fluvastatin 80 mg, pravastatin 40–80 mg, rosuvastatin 5–10 mg, and simvastatin 20–40 mg), and low-intensity (fluvastatin at 20–40 mg, pravastatin at 10–20 mg, and simvastatin at 10 mg).^1^ We also classified statins based on their lipophilicity: lipophilic statins (atorvastatin, simvastatin, lovastatin, fluvastatin, and pitavastatin) and hydrophilic statins (rosuvastatin and pravastatin). For participants who used statins of different intensities or types throughout their treatment period, classification was based on the intensity level or statin type they maintained for the longest duration.

**UKB-PPP detailed methodology**

1. Proteomic data generation and processing

The UK Biobank Pharma Proteomics Project (UKB-PPP) is a collaborative initiative involving 13 biopharmaceutical companies that generated comprehensive blood-based proteomic measurements from approximately 50,000 UK Biobank participants. Proteomic profiling was conducted using the Olink platform, a highly multiplexed immunoassay technology that enables simultaneous measurement of multiple protein biomarkers from small sample volumes. Protein levels from blood samples collected at enrollment were quantified and reported as normalized protein expression (NPX) values. Detailed methodology for the Olink technology platform and associated quality control procedures have been described in previous publications.^2^

2. Statin exposure assessment in UKB-PPP

Statin exposure in the mediation analysis cohort was determined through self-reported medication use collected via touchscreen questionnaires during the baseline assessment visit. Participants who reported regular prescription medication use were prompted to specify medication names, with additional targeted prompts specifically asking about cholesterol-lowering drugs. This medication assessment was designed to capture medications taken on a regular basis (e.g., daily, weekly, or monthly) but deliberately excluded short-term treatments, over-the-counter products, and unprescribed medications to focus on chronic therapeutic interventions.

3. Analytic Sample Construction

From the initial 53,002 UKB-PPP participants, we applied the same inclusion and exclusion criteria used in the main replication cohort, with the important distinction that eligibility was determined based on survey-reported medication data rather than prescription records. This methodological adaptation was necessary due to the different data sources available in the UKB-PPP sample. Additional exclusions specific to the mediation analysis included participants with missing COQ7 protein measurements and those reporting CoQ10 supplement use, as exogenous CoQ10 supplementation could confound the association between statin use, COQ7 levels, and dementia risk.

The mediation analysis began with 53,002 participants enrolled in the UKB-PPP. We excluded participants based on the following criteria: unknown sex at birth (n=0), insufficient survey data (n=302), non-White ethnicity (n=3,539), and missing *APOE* genotype data (n=339), resulting in a study cohort of 48,822 participants after initial enrollment criteria. From this cohort, we applied additional exclusion criteria specific to the mediation analysis. We excluded participants with dementia before the index date (n=48), neurological injury before the index date (n=1,130), follow-up duration less than 1 year (n=178), missing COQ7 protein level (n=8,691), and those taking CoQ10 supplements (n=149). These exclusions yielded a final analytic cohort of 38,553 participants. The final cohort was divided into statin non-users (n=32,445) and statin users (n=6,108) based on self-reported medication use at enrollment.

The index date was defined as the date of study enrollment. Dementia case definitions were identical to those used in the replication cohort. The observation period for each participant began at the index date and ended at the time of censoring, defined as the earliest occurrence of incident dementia, loss to follow-up, death, or study completion (May 31, 2022).

4. LDL Cholesterol Measurements

For the secondary mediation analysis examining LDL cholesterol as a potential mediator, we further restricted the analytic cohort by excluding participants with missing LDL-C measurements. LDL cholesterol concentrations were measured directly from baseline blood samples using standardized automated biochemical assays (Beckman Coulter AU5800, Brea, CA, USA) following established clinical laboratory protocols.

**Covariates**

Baseline characteristics were systematically collected including demographics (sex, age, BMI, and index year), genetic factors (*APOE* status), comorbidities, and concurrent medications, using information available at or before the index date. Demographic variables included sex, age at the index date, BMI, and index year of the index date. Sex was treated as time-invariant. BMI was defined using the measurement temporally closest to the index date. Age was categorized into four groups: <50, 50–59, 60–69, and ≥70 years. BMI was grouped into three categories: <25, 25–30, and >30 kg/m². In the mediation analysis, we additionally included smoking status and alcohol use at index date as covariates.

Comorbidities were identified from all available diagnosis records on or before the index date and included anxiety, coronary artery disease, depression, diabetes mellitus, heart failure, hypertension, kidney disease, liver disease, peripheral vascular disease, and thyroid disorders. Concomitant medication use was identified from prescription records during the 365 days preceding the index date and included benzodiazepines, antihypertensive agents, non-statin lipid-lowering agents, and tricyclic antidepressants.

The *APOE* status was extracted from genetic data in both cohorts using established genomic sequencing protocols. In the All of Us Research Program, *APOE* genotypes were obtained from short-read whole genome sequencing data following standardized bioinformatics pipelines.^3^ In the UK Biobank, genotype calls were derived from whole genome sequencing data processed through validated quality control procedures.^4^ *APOE* genetic variants were determined by examining two critical single-nucleotide variants, rs429358 and rs7412, located within the *APOE* gene on chromosome 19. These two polymorphisms define the three main *APOE* alleles: ε2, ε3, and ε4. For analytical purposes, *APOE* status was categorized based on the number of *APOE* ε4 alleles present in each participant's genotype: 0 copies (ε2/ε2, ε2/ε3, ε3/ε3), 1 copy (ε2/ε4, ε3/ε4), or 2 copies (ε4/ε4).

**Statistical analysis**

Discovery cohort analyses were performed within the All of Us Researcher Workbench using Python and R. Replication and mediation analyses used R version 4.3.1 (R Foundation for Statistical Computing, Vienna, Austria). Descriptive statistics were utilized to generate summary measures for all variables, using means and standard deviations for continuous variables and counts and percentages for categorical variables. A two-sided P-value <0.05 was considered statistically significant.

**Reference**

1. Reiter-Brennan C, Osei AD, Iftekhar Uddin SM, et al. ACC/AHA lipid guidelines: Personalized care to prevent cardiovascular disease. Cleve Clin J Med 2020;87(4):231–239.

2. Sun BB, Chiou J, Traylor M, et al. Plasma proteomic associations with genetics and health in the UK Biobank. Nature 2023;622(7982):329–338.

3. All of Us Research Program Genomics Investigators. Genomic data in the All of Us Research Program. Nature 2024;627(8003):340–346.

4. Li S, Carss KJ, Halldorsson BV, Cortes A, UK Biobank Whole-Genome Sequencing Consortium. Whole-genome sequencing of half-a-million UK Biobank participants. medRxiv. 2023.

Supplementary Table 1. Diagnostic codes used to define all-cause dementia and dementia subtypes

| Code type | Data-Coding | Coding Text | Alzheimer's disease | Vascular dementia | Dementia |
| --- | --- | --- | --- | --- | --- |
| ICD-9 | 290.2 | Senile dementia, depressed or paranoid type |  |  | ● |
| ICD-9 | 290.3 | Senile dementia with acute confusional state |  |  | ● |
| ICD-9 | 290.4 | Arteriosclerotic dementia |  | ● | ● |
| ICD-9 | 291.2 | Other alcoholic dementia |  |  | ● |
| ICD-9 | 294.1 | Dementia in other conditions classified elsewhere |  |  | ● |
| ICD-9 | 331 | Alzheimer's disease | ● |  | ● |
| ICD-9 | 331.1 | Pick's disease |  |  | ● |
| ICD-9 | 331.2 | Senile degeneration of brain |  |  | ● |
| ICD-9 | 331.5 | Creutzfeldt-Jakob disease |  |  | ● |
| ICD-10 | A81.0 | Sporadic Creutzfeldt-Jakob disease |  |  | ● |
| ICD-10 | F00 | Dementia in Alzheimer's disease | ● |  | ● |
| ICD-10 | F00.0 | Dementia in Alzheimer's disease with early onset | ● |  | ● |
| ICD-10 | F00.1 | Dementia in Alzheimer's disease with late onset | ● |  | ● |
| ICD-10 | F00.2 | Dementia in Alzheimer's disease, atypical or mixed type | ● |  | ● |
| ICD-10 | F00.9 | Dementia in Alzheimer's disease, unspecified | ● |  | ● |
| ICD-10 | F01 | Vascular dementia |  | ● | ● |
| ICD-10 | F01.0 | Vascular dementia of acute onset |  | ● | ● |
| ICD-10 | F01.1 | Multi-infarct dementia |  | ● | ● |
| ICD-10 | F01.2 | Subcortical vascular dementia |  | ● | ● |
| ICD-10 | F01.3 | Mixed cortical and sub-cortical vascular dementia |  | ● | ● |
| ICD-10 | F01.8 | Other vascular dementia |  | ● | ● |
| ICD-10 | F01.9 | Vascular dementia, unspecified |  | ● | ● |
| ICD-10 | F02 | Dementia in other diseases classified elsewhere |  |  | ● |
| ICD-10 | F02.0 | Dementia in Picks disease |  |  | ● |
| ICD-10 | F02.1 | Dementia in Creutzfeldt-Jacob disease |  |  | ● |
| ICD-10 | F02.2 | Dementia in Huntington’s disease |  |  | ● |
| ICD-10 | F02.3 | Dementia in Parkinson’s disease |  |  | ● |
| ICD-10 | F02.4 | Dementia in HIV disease |  |  | ● |
| ICD-10 | F02.8 | Dementia in other specified diseases classified elsewhere |  |  | ● |
| ICD-10 | F03 | Unspecified dementia |  |  | ● |
| ICD-10 | F05.1 | Delirium superimposed on dementia |  |  | ● |
| ICD-10 | F10.6 | Mental and behavioral disorders due to use of alcohol - amnesic syndrome |  |  | ● |
| ICD-10 | G30 | Alzheimer’s disease | ● |  | ● |
| ICD-10 | G30.0 | Alzheimer’s disease with early onset | ● |  | ● |
| ICD-10 | G30.1 | Alzheimer’s disease with late onset | ● |  | ● |
| ICD-10 | G30.8 | Other Alzheimer's disease | ● |  | ● |
| ICD-10 | G30.9 | Alzheimer's disease unspecified | ● |  | ● |
| ICD-10 | G31.0 | Circumscribed brain atrophy |  |  | ● |
| ICD-10 | G31.1 | Senile degeneration of brain |  |  | ● |
| ICD-10 | G31.8 | Other specified degenerative diseases of nervous system |  |  | ● |
| ICD-10 | I67.3 | Binswanger’s disease |  | ● | ● |
| SNOMED | 26929004 | Alzheimer's disease | ● | ● | ● |
| SNOMED | 135761000119101 | Cerebral degeneration due to alcoholism |  |  | ● |
| SNOMED | 792004 | Creutzfeldt-Jakob disease |  |  | ● |
| SNOMED | 52522001 | Degenerative brain disorder |  |  | ● |
| SNOMED | 52448006 | Dementia |  |  | ● |
| SNOMED | 281004 | Dementia associated with alcoholism |  |  | ● |
| SNOMED | 191519005 | Dementia associated with another disease |  |  | ● |
| SNOMED | 1591000119103 | Dementia with behavioral disturbance |  |  | ● |
| SNOMED | 80098002 | Diffuse Lewy body disease |  |  | ● |
| SNOMED | 191493005 | Drug-induced dementia |  |  | ● |
| SNOMED | 230270009 | Frontotemporal dementia |  |  | ● |
| SNOMED | 428051000124108 | Mild dementia |  |  | ● |
| SNOMED | 110352000 | Minimal cognitive impairment |  |  | ● |
| SNOMED | 430771000124100 | Moderate dementia |  |  | ● |
| SNOMED | 10349009 | Multi-infarct dementia with delirium |  | ● | ● |
| SNOMED | 25772007 | Multi-infarct dementia with delusions |  | ● | ● |
| SNOMED | 14070001 | Multi-infarct dementia with depression |  | ● | ● |
| SNOMED | 70936005 | Multi-infarct dementia, uncomplicated |  | ● | ● |
| SNOMED | 191452002 | Presenile dementia with delirium |  |  | ● |
| SNOMED | 191455000 | Presenile dementia with depression |  |  | ● |
| SNOMED | 416780008 | Primary degenerative dementia of the Alzheimer type, presenile onset | ● | ● | ● |
| SNOMED | 416975007 | Primary degenerative dementia of the Alzheimer type, senile onset | ● | ● | ● |
| SNOMED | 111480006 | Psychoactive substance-induced organic dementia |  |  | ● |
| SNOMED | 45864009 | Senile degeneration of brain |  |  | ● |
| SNOMED | 312991009 | Senile dementia of the Lewy body type |  |  | ● |
| SNOMED | 191461002 | Senile dementia with delirium |  |  | ● |
| SNOMED | 371024007 | Senile dementia with delusion |  |  | ● |
| SNOMED | 191459006 | Senile dementia with depression |  |  | ● |
| SNOMED | 428351000124105 | Severe dementia |  |  | ● |
| SNOMED | 191451009 | Uncomplicated presenile dementia |  |  | ● |
| SNOMED | 191449005 | Uncomplicated senile dementia |  |  | ● |
| SNOMED | 429998004 | Vascular dementia |  | ● | ● |
| SNOMED | 288631000119104 | Vascular dementia with behavioral disturbance |  | ● | ● |
| SNOMED | 16276361000119109 | Vascular dementia without behavioral disturbance |  | ● | ● |
| Read | E002. | Senile dementia with depressive or paranoid features |  |  | ● |
| Read | E0020 | Senile dementia with paranoia |  |  | ● |
| Read | E0021 | Senile dementia with depression |  |  | ● |
| Read | E002z | Senile dementia with depressive or paranoid features NOS |  |  | ● |
| Read | E003. | Senile dementia with delirium |  |  | ● |
| Read | E004. | Arteriosclerotic dementia |  | ● | ● |
| Read | E0040 | Uncomplicated arteriosclerotic dementia |  | ● | ● |
| Read | E0041 | Arteriosclerotic dementia with delirium |  | ● | ● |
| Read | E0042 | Arteriosclerotic dementia with paranoia |  | ● | ● |
| Read | E0043 | Arteriosclerotic dementia with depression |  | ● | ● |
| Read | E004z | Arteriosclerotic dementia NOS |  | ● | ● |
| Read | E012. | Other alcoholic dementia |  |  | ● |
| Read | Eu00z | [X]Dementia in Alzheimer's disease, unspecified | ● |  | ● |
| Read | Eu01. | [X]Vascular dementia |  | ● | ● |
| Read | Eu010 | [X]Vascular dementia of acute onset |  | ● | ● |
| Read | Eu011 | [X]Multi-infarct dementia |  | ● | ● |
| Read | Eu012 | [X]Subcortical vascular dementia |  | ● | ● |
| Read | Eu013 | [X]Mixed cortical and subcortical vascular dementia |  | ● | ● |
| Read | Eu01y | [X]Other vascular dementia |  | ● | ● |
| Read | Eu01z | [X]Vascular dementia, unspecified |  | ● | ● |
| Read | Eu02. | [X]Dementia in other diseases classified elsewhere |  |  | ● |
| Read | Eu02z | [X] Unspecified dementia |  |  | ● |
| Read | Eu041 | [X]Delirium superimposed on dementia |  |  | ● |
| Read | F11.. | Other cerebral degenerations |  |  | ● |
| Read | F110. | Alzheimer's disease | ● |  | ● |
| Read | F1100 | Alzheimer's disease with early onset | ● |  | ● |
| Read | F1101 | Alzheimer's disease with late onset | ● |  | ● |
| Read | F111. | Pick's disease |  |  | ● |
| Read | F112. | Senile degeneration of brain |  |  | ● |
| Read | F118. | Frontotemporal degeneration |  |  | ● |
| Read | F11y. | Other cerebral degeneration |  |  | ● |
| Read | F11y2 | Corticobasal degeneration |  |  | ● |
| Read | F11yz | Other cerebral degeneration NOS |  |  | ● |
| Read | F21y2 | Binswanger's disease |  | ● | ● |
| Read | Fyu30 | [X]Other Alzheimer's disease | ● |  | ● |
| Read | Fyu31 | [X]Other specified degenerative diseases of the nervous system |  |  | ● |

Abbreviations: ICD, International Classification of Diseases.

Supplementary Table 2. Read codes and drug names used to identify statin prescriptions in the UK Biobank

| Data field | Type | Name | Drug name | Read v2 code |
| --- | --- | --- | --- | --- |
| 42039 | Simvastatins | Simvastatin | EZETIMIBE + SIMVASTATIN tabs 10mg + 20mg, EZETIMIBE + SIMVASTATIN tabs 10mg + 40mg, EZETIMIBE + SIMVASTATIN tabs 10mg + 80mg, Ezetimibe And Simvastatin Tablets 10 mg + 20 mg, Ezetimibe And Simvastatin Tablets 10 mg + 40 mg, Ezetimibe And Simvastatin Tablets 10 mg + 20 mg, SIMVASTATIN, Simvastatin Oral Suspension (Sugar-Free) 40 mg/5 ml, Simvastatin Tablets 10 mg, Simvastatin Tablets 20 mg, Simvastatin Tablets 40 mg, Simvastatin Tablets 80 mg, SIMVASTATIN + EZETIMIBE tabs 20mg + 10mg, SIMVASTATIN + EZETIMIBE tabs 40mg + 10mg, SIMVASTATIN 10 MG TAB, Simvastatin 10 mg tablets, Simvastatin 10mg, SIMVASTATIN 10mg tablets, Simvastatin 10mg tablets (A A H Pharmaceuticals Ltd), Simvastatin 10mg tablets (Actavis UK Ltd), Simvastatin 10mg tablets (Almus Pharmaceuticals Ltd), Simvastatin 10mg tablets (Dexcel-Pharma Ltd), Simvastatin 10mg tablets (Kent Pharmaceuticals Ltd), Simvastatin 10mg tablets (Mylan Ltd), Simvastatin 10mg tablets (Teva UK Ltd), SIMVASTATIN 10MG TABS, SIMVASTATIN 20 MG TAB, Simvastatin 20 mg tablets, SIMVASTATIN 20MG, Simvastatin 20mg / Ezetimibe 10mg tablets, SIMVASTATIN 20mg tablets, Simvastatin 20mg tablets (A A H Pharmaceuticals Ltd), Simvastatin 20mg tablets (Actavis UK Ltd), Simvastatin 20mg tablets (Almus Pharmaceuticals Ltd), Simvastatin 20mg tablets (Kent Pharmaceuticals Ltd), Simvastatin 20mg tablets (Mylan Ltd), Simvastatin 20mg tablets (PLIVA Pharma Ltd), Simvastatin 20mg tablets (Sandoz Ltd), Simvastatin 20mg tablets (Teva UK Ltd), Simvastatin 20mg tablets (Wockhardt UK Ltd), Simvastatin 20mg with ezetimibe 10mg tablet, SIMVASTATIN 20MG. TABLETS, SIMVASTATIN 20mg/5mL oral suspension, Simvastatin 20mg/5ml oral suspension sugar free, Simvastatin 20mg/5ml oral suspension sugar free (A A H Pharmaceuticals Ltd), Simvastatin 20mg/5ml oral suspension sugar free (Rosemont Pharmaceuticals Ltd), SIMVASTATIN 20mg/EZETIMIBE 10mg tablets, SIMVASTATIN 40 MG TAB, Simvastatin 40 mg tablets, Simvastatin 40mg / Ezetimibe 10mg tablets, SIMVASTATIN 40MG 40MG, SIMVASTATIN 40MG TAB, SIMVASTATIN 40MG TABLET, SIMVASTATIN 40mg tablets, Simvastatin 40mg tablets (A A H Pharmaceuticals Ltd), Simvastatin 40mg tablets (Actavis UK Ltd), Simvastatin 40mg tablets (Almus Pharmaceuticals Ltd), Simvastatin 40mg tablets (Dexcel-Pharma Ltd), Simvastatin 40mg tablets (Kent Pharmaceuticals Ltd), Simvastatin 40mg tablets (Mylan Ltd), Simvastatin 40mg tablets (Ranbaxy (UK) Ltd), Simvastatin 40mg tablets (Sandoz Ltd), Simvastatin 40mg tablets (Teva UK Ltd), Simvastatin 40mg tablets (Wockhardt UK Ltd), Simvastatin 40mg with ezetimibe 10mg tablet, SIMVASTATIN 40mg/5mL oral suspension, Simvastatin 40mg/5ml oral suspension sugar free, Simvastatin 40mg/5ml oral suspension sugar free (A A H Pharmaceuticals Ltd), Simvastatin 40mg/5ml oral suspension sugar free (Rosemont Pharmaceuticals Ltd), SIMVASTATIN 40mg/EZETIMIBE 10mg tablets, SIMVASTATIN 40MGS TABS, Simvastatin 80mg / Ezetimibe 10mg tablets, SIMVASTATIN 80mg tablets, Simvastatin 80mg tablets (A A H Pharmaceuticals Ltd), Simvastatin 80mg tablets (Actavis UK Ltd), Simvastatin 80mg tablets (Almus Pharmaceuticals Ltd), Simvastatin 80mg tablets (Focus Pharmaceuticals Ltd), Simvastatin 80mg tablets (IVAX Pharmaceuticals UK Ltd), Simvastatin 80mg tablets (Mylan Ltd), Simvastatin 80mg tablets (Teva UK Ltd), SIMVASTATIN 80mg/EZETIMIBE 10mg tablets, SIMVASTATIN oral liq 40mg/5ml, simvastatin oral suspension sugar-free 20mg/5ml, SIMVASTATIN sf oral susp 40mg/5ml, SIMVASTATIN TAB 10, SIMVASTATIN TAB 10mg, SIMVASTATIN TAB 20, SIMVASTATIN TAB 20mg, SIMVASTATIN TAB 40, SIMVASTATIN TAB 40mg, Simvastatin Tablets 10 mg, simvastatin tablets 10mg, SIMVASTATIN TABLETS 10MG-P42 0, Simvastatin Tablets 20 mg, simvastatin tablets 20mg, SIMVASTATIN TABLETS 20MG-P42 0, Simvastatin Tablets 40 mg, simvastatin tablets 40mg, Simvastatin Tablets 80 mg, simvastatin tablets 80mg, SIMVASTATIN tabs 10 mg, Simvastatin TABS 10MG, SIMVASTATIN tabs 20 mg, Simvastatin TABS 20MG, SIMVASTATIN tabs 40 mg, Simvastatin TABS 40mg, Simvastatin TABS 80MG, Simvastatin-P42 10mg Tablet | bxdA.  bxdB.  bxdC.  bxdK.  bxd..  bxd1.  bxd2.  bxdu.  bxdy.  bxd5.  bxdv.  bxdx.  bxdz.  bxdw. |
|  |  | Zocor | *ZOCOR HEART-PRO 10mg tablets, ZOCOR, Zocor Tablets 40 mg, ZOCOR 10mg tablets, Zocor 10mg tablets (Merck Sharp & Dohme Ltd), ZOCOR 10mg tablets 28CP, ZOCOR 20mg tablets, Zocor 20mg tablets (Merck Sharp & Dohme Ltd), ZOCOR 20mg tablets 28CP, ZOCOR 40mg tablets, Zocor 40mg tablets (Merck Sharp & Dohme Ltd), ZOCOR 80mg tablets, Zocor 80mg tablets (Merck Sharp & Dohme Ltd), ZOCOR TAB 10, ZOCOR TAB 10mg, ZOCOR TAB 20, ZOCOR TAB 20mg, ZOCOR TAB 40mg, Zocor Tablets 10 mg, Zocor TABLETS 10MG, ZOCOR tablets 20mg, Zocor TABS 10MG, Zocor TABS 20MG, Zocor-P42 10mg Tablet | bxdG.  bxd7.  bxd3.  bxd8.  bxd4.  bxd6.  bxd9. |
|  |  | Simvador | Simvador Tablets 10 mg, Simvador Tablets 20 mg, Simvador Tablets 40 mg, Simvador Tablets 80 mg, SIMVADOR 10mg tablets, Simvador 10mg tablets (Discovery Pharmaceuticals Ltd), Simvador 10mg tablets (Discovery Pharmaceuticals), SIMVADOR 20mg tablets, Simvador 20mg tablets (Discovery Pharmaceuticals Ltd), Simvador 20mg tablets (Discovery Pharmaceuticals), SIMVADOR 40mg tablets, Simvador 40mg tablets (Discovery Pharmaceuticals Ltd), Simvador 40mg tablets (Discovery Pharmaceuticals), SIMVADOR 80mg tablets, Simvador 80mg tablets (Discovery Pharmaceuticals Ltd), Simvador 80mg tablets (Discovery Pharmaceuticals), SIMVADOR TABLETS 40MG, SIMVADOR tabs 10mg, SIMVADOR tabs 20mg, Simvador TABS 40 mg, SIMVADOR tabs 40mg | bxdA.  bxdB.  bxdC.  bxdK. |
|  |  | Ranzolont | *RANZOLONT 10mg tablets, *RANZOLONT 20mg tablets, *RANZOLONT 40mg tablets | bxdD.  bxdE.  bxdF. |
|  |  | Inegy | Inegy Tablets 10 mg/20 mg, Inegy Tablets 10 mg/40 mg, Inegy Tablets 10 mg/80 mg, INEGY 10mg/20mg tablets, Inegy 10mg/20mg tablets (Merck Sharp & Dohme Ltd), INEGY 10mg/40mg tablets, Inegy 10mg/40mg tablets (Merck Sharp & Dohme Ltd), Inegy 10mg/40mg tablets (MSD-SP Ltd), Inegy 10mg/40mg TABS, INEGY 10mg/80mg tablets, Inegy 10mg/80mg tablets (Merck Sharp & Dohme Ltd), INEGY tabs 10mg + 20mg, INEGY tabs 10mg + 40mg | bxdH.  bxdI.  bxdJ. |
|  | Rosuvastatins | Rosuvastatin | ROSUVASTATIN, Rosuvastatin Tablets 10 mg, Rosuvastatin Tablets 20 mg, Rosuvastatin Tablets 40 mg, Rosuvastatin Tablets 5 mg, Rosuvastatin 10 mg tablets, ROSUVASTATIN 10MG, ROSUVASTATIN 10mg tablets, ROSUVASTATIN 20mg tablets, Rosuvastatin 40 mg tablets, ROSUVASTATIN 40mg tablets, ROSUVASTATIN 5mg tablets, Rosuvastatin Tablets 10 mg, rosuvastatin tablets 10mg, ROSUVASTATIN TABLETS 20 MG, rosuvastatin tablets 20mg, rosuvastatin tablets 40mg, Rosuvastatin Tablets 5 mg, rosuvastatin tablets 5mg, Rosuvastatin TABS 10mg, Rosuvastatin TABS 20MG, Rosuvastatin TABS 40MG, Rosuvastatin TABS 5mg | bxk..  bxkx.  bxky.  bxkz.  bxkw. |
|  |  | Crestor | Crestor Tablets 5 mg, CRESTOR 10mg tablets, Crestor 10mg tablets (AstraZeneca UK Ltd), CRESTOR 20mg tablets, Crestor 20mg tablets (AstraZeneca UK Ltd), CRESTOR 40mg tablets, Crestor 40mg tablets (AstraZeneca UK Ltd), CRESTOR 5mg tablets, Crestor 5mg tablets (AstraZeneca UK Ltd), CRESTOR TABLETS 10MG, CRESTOR tabs 10mg, CRESTOR tabs 20mg, CRESTOR tabs 40mg, CRESTOR tabs 5mg | bxk1.  bxk2.  bxk3.  bxk4. |
|  | Pravastatins | Pravastatin | *PRAVASTATIN 10mg tablets, *PRAVASTATIN 20mg tablets, PRAVASTATIN, Pravastatin Tablets 10 mg, Pravastatin Tablets 20 mg, Pravastatin Tablets 40 mg, Pravastatin 10mg tablets, Pravastatin 10mg tablets (A A H Pharmaceuticals Ltd), Pravastatin 10mg tablets (Actavis UK Ltd), Pravastatin 10mg tablets (Almus Pharmaceuticals Ltd), Pravastatin 10mg tablets (Mylan Ltd), Pravastatin 10mg tablets (Teva UK Ltd), PRAVASTATIN 20MG, Pravastatin 20mg tablets, Pravastatin 20mg tablets (A A H Pharmaceuticals Ltd), Pravastatin 20mg tablets (Almus Pharmaceuticals Ltd), Pravastatin 20mg tablets (Mylan Ltd), Pravastatin 20mg tablets (Sandoz Ltd), Pravastatin 20mg tablets (Teva UK Ltd), PRAVASTATIN 20MG TABS, PRAVASTATIN 40MG, Pravastatin 40mg tablets, Pravastatin 40mg tablets (A A H Pharmaceuticals Ltd), Pravastatin 40mg tablets (Actavis UK Ltd), Pravastatin 40mg tablets (Almus Pharmaceuticals Ltd), Pravastatin 40mg tablets (Mylan Ltd), Pravastatin 40mg tablets (Teva UK Ltd), PRAVASTATIN SODIUM, PRAVASTATIN SODIUM 10mg tablets, PRAVASTATIN SODIUM 10mg tabs, PRAVASTATIN SODIUM 20mg tablets, Pravastatin sodium 40 mg tablets, PRAVASTATIN SODIUM 40mg tablets, PRAVASTATIN SODIUM 40mg tabs, Pravastatin Sodium TABLETS 10MG, PRAVASTATIN SODIUM TABLETS 20MG, PRAVASTATIN SODIUM TABLETS 40MG, Pravastatin Sodium TABS 10MG, Pravastatin Sodium TABS 20MG, Pravastatin Sodium TABS 40MG, PRAVASTATIN TAB 10, PRAVASTATIN TAB 10mg, PRAVASTATIN TAB 20, PRAVASTATIN TAB 20mg, Pravastatin Tablets 10 mg, PRAVASTATIN tablets 10mg, PRAVASTATIN TABLETS 10MG-P42 0, Pravastatin Tablets 20 mg, pravastatin tablets 20mg, PRAVASTATIN TABLETS 20MG-P42 0, Pravastatin Tablets 40 mg, pravastatin tablets 40mg, PRAVASTATIN TABS, PRAVASTATIN tabs 10 MG, PRAVASTATIN tabs 10mg, PRAVASTATIN tabs 20mg, PRAVASTATIN TABS 40 MG, PRAVASTATIN tabs 40mg | bxe..  bxe1.  bxe2.  bxe5.  bxe6.  bxe7. |
|  |  | Lipostat | LIPOSTAT 10mg tablets, Lipostat 10mg tablets (Bristol-Myers Squibb Pharmaceuticals Ltd), LIPOSTAT 20mg tablets, Lipostat 20mg tablets (Bristol-Myers Squibb Pharmaceuticals Ltd), LIPOSTAT 40mg tablets, Lipostat 40mg tablets (Bristol-Myers Squibb Pharmaceuticals Ltd), LIPOSTAT TAB 10, LIPOSTAT TAB 10mg, LIPOSTAT TAB 20mg, LIPOSTAT TABLETS 10MG, Lipostat TABS 10MG, Lipostat TABS 20MG, Lipostat TABS 40MG | bxe3.  bxe4.  bxe8. |
|  | Fluvastatins | Fluvastatin | Fluvastatin Capsules 20 mg, Fluvastatin Capsules 40 mg, FLUVASTATIN 20mg capsules, Fluvastatin 20mg capsules (A A H Pharmaceuticals Ltd), Fluvastatin 20mg capsules (Actavis UK Ltd), fluvastatin 24 hour modified release tablets 80mg, FLUVASTATIN 24 hr mr tabs 80mg, FLUVASTATIN 40mg capsules, Fluvastatin 40mg capsules (A A H Pharmaceuticals Ltd), FLUVASTATIN 80mg m/r tablets, Fluvastatin 80mg modified-release tablets, FLUVASTATIN CAP 20, FLUVASTATIN CAP 40, Fluvastatin CAPS 20MG, FLUVASTATIN caps 40 mg, Fluvastatin CAPS 40MG, Fluvastatin Capsules 20 mg, fluvastatin capsules 20mg, Fluvastatin Capsules 40 mg, fluvastatin capsules 40mg, FLUVASTATIN MR TABLETS 80MG, FLUVASTATIN SODIUM | bxg..  bxg3.  bxg4.  bxgz. |
|  |  | Lescol | LESCOL 20mg capsules, Lescol 20mg capsules (Novartis Pharmaceuticals UK Ltd), LESCOL 40mg capsules, Lescol 40mg capsules (Novartis Pharmaceuticals UK Ltd), LESCOL CAPSULES 20MG, LESCOL CAPSULES 40MG, LESCOL XL 80mg m/r tablets, Lescol XL 80mg tablets (Novartis Pharmaceuticals UK Ltd), LESCOL XL tabs 80mg | bxg1.  bxg2.  bxg5. |
|  |  | Luvinsta | LUVINSTA XL 80mg m/r tablets | bxg6. |
|  |  | Stefluvin | STEFLUVIN XL 80mg m/r tablets | bxg7. |
|  |  | Dorisin | DORISIN XL 80mg m/r tablets | bxg8. |
|  |  | Nandovar | NANDOVAR XL 80mg m/r tablets | bxg9. |
|  | Atorvastatins | Atorvastatin | *ATORVASTATIN - -, ATORVASTATIN, Atorvastatin Chewable Tablets Sugar Free 10 mg, Atorvastatin Tablets 10 mg, Atorvastatin Tablets 10 mg~(bxi1.), Atorvastatin Tablets 20 mg, Atorvastatin Tablets 40 mg, Atorvastatin Tablets 80 mg, ATORVASTATIN 10 MG, Atorvastatin 10 mg tablets, ATORVASTATIN 10 mg tabs, ATORVASTATIN 10 mg tabs -, ATORVASTATIN 10MG, ATORVASTATIN 10mg chewable tablets, Atorvastatin 10mg chewable tablets sugar free, ATORVASTATIN 10MG TAB, ATORVASTATIN 10mg tablets, Atorvastatin 10mg tablets (Actavis UK Ltd), Atorvastatin 10mg tablets (Almus Pharmaceuticals Ltd), Atorvastatin 10mg tablets (Dr Reddy's Laboratories (UK) Ltd), Atorvastatin 10mg tablets (Teva UK Ltd), ATORVASTATIN 10MGS TABS, ATORVASTATIN 20 MG TABS, ATORVASTATIN 20MG, ATORVASTATIN 20mg chewable tablets, Atorvastatin 20mg chewable tablets sugar free, ATORVASTATIN 20MG TAB, ATORVASTATIN 20mg tablets, Atorvastatin 20mg tablets (Actavis UK Ltd), Atorvastatin 20mg tablets (Aspire Pharma Ltd), Atorvastatin 20mg tablets (Teva UK Ltd), Atorvastatin 20mg tablets (Wockhardt UK Ltd), Atorvastatin 20mg/5ml oral suspension, ATORVASTATIN 20MGS, Atorvastatin 30mg tablets, ATORVASTATIN 40 MG TABS, ATORVASTATIN 40MG, ATORVASTATIN 40MG TAB, ATORVASTATIN 40mg tablets, Atorvastatin 40mg tablets (Actavis UK Ltd), Atorvastatin 40mg tablets (Aspire Pharma Ltd), Atorvastatin 40mg tablets (Teva UK Ltd), Atorvastatin 40mg tablets (Wockhardt UK Ltd), ATORVASTATIN 60mg tablets, ATORVASTATIN 80MG TABLET, ATORVASTATIN 80mg tablets, Atorvastatin 80mg tablets (Actavis UK Ltd), Atorvastatin 80mg tablets (Almus Pharmaceuticals Ltd), Atorvastatin 80mg tablets (Teva UK Ltd), ATORVASTATIN TAB 10, ATORVASTATIN TAB 10mg, ATORVASTATIN TAB 20, ATORVASTATIN TAB 20mg, ATORVASTATIN TAB 40mg, Atorvastatin Tablets 10 mg, atorvastatin tablets 10mg, Atorvastatin Tablets 20 mg, atorvastatin tablets 20mg, Atorvastatin Tablets 40 mg, atorvastatin tablets 40mg, Atorvastatin Tablets 80 mg, Atorvastatin Tablets 80mg, Atorvastatin TABS 10MG, atorvastatin tabs 20mg, Atorvastatin TABS 40MG, Atorvastatin TABS 80MG | bxi..  bxi9.  bxi1.  bxiB.  bxi2.  bxi3.  bxiy.  bxiz. |
|  |  | Lipitor | *LIPITOR TABS 10 MG, Lipitor Tablets 10 mg, Lipitor Tablets 20 mg, Lipitor Tablets 40 mg, Lipitor Tablets 80 mg, LIPITOR 10mg chewable tablets, Lipitor 10mg chewable tablets (Pfizer Ltd), LIPITOR 10mg tablets, Lipitor 10mg tablets (Pfizer Ltd), LIPITOR 10MG TABS, LIPITOR 20mg chewable tablets, Lipitor 20mg chewable tablets (Pfizer Ltd), LIPITOR 20mg tablets, Lipitor 20mg tablets (Pfizer Ltd), LIPITOR 40mg tablets, Lipitor 40mg tablets (Pfizer Ltd), LIPITOR 80mg tablets, Lipitor 80mg tablets (Pfizer Ltd), LIPITOR TAB 10mg, Lipitor Tablets 10 mg, LIPITOR tablets 10mg, Lipitor TABS 10MG, Lipitor TABS 20MG, LIPITOR tabs 40mg | bxi8.  bxi4.  bxiA.  bxi5.  bxi6.  bxi7. |

Supplementary Table 3. Medication codes used to identify statin use in the UK Biobank based on baseline survey responses

| Data field | Type | Name | Data-coding |
| --- | --- | --- | --- |
| 20003 | Simvastatins | Simvastatin | 1140861958 |
|  |  | Zocor | 1140881748 |
|  |  | Simvador | 1141188146 |
|  | Rosuvastatins | Rosuvastatin | 1141192410 |
|  |  | Crestor | 1141192414 |
|  | Pravastatins | Pravastatin | 1140888648 |
|  |  | Lipostat | 1140861970 |
|  | Fluvastatins | Fluvastatin | 1140888594 |
|  |  | Lescol | 1140864592 |
|  | Atorvastatins | Atorvastatin | 1141146234 |
|  |  | Lipitor | 1141146138 |

Supplementary Table 4. Baseline characteristics of statin users and non-users before and after inverse probability of treatment weighting in the discovery cohort.

|  | Before IPTW | | | After IPTW | | |
| --- | --- | --- | --- | --- | --- | --- |
|  | Statin non-user (n=51,842) | Statin user (n=31,513) | SMD | Statin non-user (n=83,440.5) | Statin user (n=81,665.2) | SMD |
| Sex at birth |  |  | 0.280 |  |  | 0.021 |
| Male | 19,824 (38.2) | 16,394 (52.0) |  | 35,829.6 (42.9) | 35,914.8 (44.0) |  |
| Female | 32,018 (61.8) | 15,119 (48.0) |  | 47,610.9 (57.1) | 45,750.4 (56.0) |  |
| Age (year) |  |  | 0.262 |  |  | 0.051 |
| <50 | 4,108 (7.9) | 1,119 (3.6) |  | 5,001.4 (6.0) | 4,087.8 (5.0) |  |
| 50–59 | 17,697 (34.1) | 8,644 (27.4) |  | 25,351.9 (30.4) | 24,053.2 (29.5) |  |
| 60–69 | 19,143 (36.9) | 13,892 (44.1) |  | 32,726.6 (39.2) | 33,095.8 (40.5) |  |
| ≥70 | 10,894 (21.0) | 7,858 (24.9) |  | 20,360.6 (24.4) | 20,428.5 (25.0) |  |
| Index year |  |  | 0.141 |  |  | 0.026 |
| 2006–2009 | 9,057 (17.5) | 6,486 (20.6) |  | 13,674.3 (16.4) | 13,578.8 (16.6) |  |
| 2010–2014 | 15,016 (29.0) | 10,182 (32.3) |  | 23,894.0 (28.6) | 23,217.3 (28.4) |  |
| 2015–2019 | 19,427 (37.5) | 10,893 (34.6) |  | 32,085.7 (38.5) | 30,698.0 (37.6) |  |
| 2020–2023 | 8,342 (16.1) | 3,952 (12.5) |  | 13,786.5 (16.5) | 14,171.1 (17.4) |  |
| BMI (kg/m^2^) |  |  | 0.337 |  |  | 0.022 |
| <25 | 17,947 (34.6) | 6,483 (20.6) |  | 24,408.3 (29.3) | 23,067.5 (28.2) |  |
| 25–30 | 17,476 (33.7) | 11,308 (35.9) |  | 28,796.2 (34.5) | 28,575.7 (35.0) |  |
| >30 | 16,419 (31.7) | 13,722 (43.5) |  | 30,236.0 (36.2) | 30,022.0 (36.8) |  |
| *APOE* status |  |  | 0.063 |  |  | 0.006 |
| No ε4 allele | 39,705 (76.6) | 23,275 (73.9) |  | 63,117.8 (75.6) | 61,619.7 (75.5) |  |
| 1 ε4 Allele | 11,345 (21.9) | 7,686 (24.4) |  | 18,950.3 (22.7) | 18,731.2 (22.9) |  |
| 2 ε4 Allele | 792 (1.5) | 552 (1.8) |  | 1,372.5 (1.6) | 1,314.3 (1.6) |  |
| Comorbidity |  |  |  |  |  |  |
| Anxiety | 3,549 (6.8) | 3,756 (11.9) | 0.175 | 8,345.4 (10.0) | 8,331.9 (10.2) | 0.007 |
| Coronary artery disease | 1,852 (3.6) | 5,521 (17.5) | 0.466 | 8,079.9 (9.7) | 7,706.1 (9.4) | 0.008 |
| Depression | 4,838 (9.3) | 5,942 (18.9) | 0.276 | 11,962.2 (14.3) | 11,706.3 (14.3) | <0.001 |
| Diabetes mellitus | 1,928 (3.7) | 5,794 (18.4) | 0.481 | 8,389.0 (10.1) | 8,057.1 (9.9) | 0.006 |
| Heart failure | 717 (1.4) | 1,408 (4.5) | 0.184 | 2,559.0 (3.1) | 2,358.4 (2.9) | 0.011 |
| Hypertension | 8,370 (16.1) | 15,603 (49.5) | 0.760 | 2,4766.2 (29.7) | 24,403.4 (29.9) | 0.004 |
| Kidney disease | 1,301 (2.5) | 1,883 (6.0) | 0.173 | 3,832.4 (4.6) | 3,670.0 (4.5) | 0.005 |
| Liver disease | 939 (1.8) | 1,115 (3.5) | 0.107 | 2,256.1 (2.7) | 2,240.5 (2.7) | 0.002 |
| Peripheral vascular disease | 573 (1.1) | 1,076 (3.4) | 0.156 | 1,946.3 (2.3) | 1,834.0 (2.2) | 0.006 |
| Thyroid disorder | 5,288 (10.2) | 5,698 (18.1) | 0.228 | 12,334.5 (14.8) | 12,111.9 (14.8) | 0.001 |
| Medication |  |  |  |  |  |  |
| Benzodiazepines | 7,413 (14.3) | 7,413 (23.5) | 0.237 | 16,234.8 (19.5) | 16,157.6 (19.8) | 0.008 |
| Blood pressure medications | 10,897 (21.0) | 14,422 (45.8) | 0.544 | 26,526.3 (31.8) | 26,380.5 (32.3) | 0.011 |
| Other lipid lowering agents | 636 (1.2) | 1,474 (4.7) | 0.205 | 2,237.1 (2.7) | 2,206.6 (2.7) | 0.001 |
| Tricyclic antidepressants | 1,005 (1.9) | 1,270 (4.0) | 0.123 | 2,487.8 (3.0) | 2,486.5 (3.0) | 0.004 |

Values are expressed as number (%). SMD < 0.1 indicates negligible imbalance.

Abbreviations: APOE, apolipoprotein E; BMI, body mass index; IPTW: inverse probability of treatment weighting; SMD: standardized mean difference.

Supplementary Table 5. Association between statin type and dementia risk in the discovery cohort.

|  | Case | Control | Person-years | Incidence rate† | HR (95% CI) | |
| --- | --- | --- | --- | --- | --- | --- |
|  |  |  |  |  | Unadjusted | Adjusted‡ |
| Statin non-user | 2,148 | 81,292 | 699,981.3 | 30.7 | 1 (Ref) | 1 (Ref) |
| Statin user |  |  |  |  |  |  |
| Lipophilic statin | 2,892 | 59,619 | 462,490.7 | 62.5 | 2.11 (1.91–2.32)*** | 2.14 (1.94–2.36)*** |
| Hydrophilic statin | 928 | 18,227 | 126,147.7 | 73.6 | 2.58 (2.25–2.96)*** | 2.50 (2.18–2.87)*** |

Data are derived from weighted proportions and are not absolute numbers. Abbreviations: CI, confidence interval; HR, hazard ratio.

† Incidence rate per 10,000 person-years. ‡ Adjusted for sex, age groups, index year, BMI categories, *APOE* status, comorbidities and medications.

**P* <0.05, ***P* <0.01, ****P* <0.001

Supplementary Table 6. Comparison of dementia risk by statin type within statin intensity groups in the discovery cohort.

| Statin user | Case | Control | Person-years | Incidence rate† | HR (95% CI) | |
| --- | --- | --- | --- | --- | --- | --- |
|  |  |  |  |  | Unadjusted | Adjusted‡ |
| High intensity |  |  |  |  |  |  |
| Lipophilic statin | 967 | 15,724 | 115,830.2 | 83.4 | 1 (Ref) | 1 (Ref) |
| Hydrophilic statin | 361 | 6,521 | 41,202.9 | 87.7 | 1.09 (0.88–1.36) | 1.10 (0.88–1.37) |
| moderate-to-low intensity |  |  |  |  |  |  |
| Lipophilic statin | 1,729 | 38,424 | 304,704.6 | 56.7 | 1 (Ref) | 1 (Ref) |
| Hydrophilic statin | 492 | 9,890 | 71,506.4 | 68.8 | 1.28 (1.08–1.52)** | 1.15 (0.97–1.37) |

Data are derived from weighted proportions and are not absolute numbers. Abbreviations: CI, confidence interval; HR, hazard ratio.

† Incidence rate per 10,000 person-years. ‡ Adjusted for sex, age groups, index year, BMI categories, *APOE* status, comorbidities and medications.

**P* <0.05, ***P* <0.01, ****P* <0.001

Supplementary Table 7. Baseline characteristics of statin users and non-users before and after inverse probability of treatment weighting in the replication cohort.

|  | Before IPTW | | | After IPTW | | |
| --- | --- | --- | --- | --- | --- | --- |
| Characteristics | Statin non-user (n=129,306) | Statin user (n=43,976) | SMD | Statin non-user (n=176,103.9) | Statin user (n=167,265.5) | SMD |
| Sex at birth |  |  | 0.356 |  |  | 0.015 |
| Male | 50,275 (38.9) | 24,794 (56.4) |  | 76484.0 (43.4) | 71401.2 (42.7) |  |
| Female | 79031 (61.1) | 19182 (43.6) |  | 99619.9 (56.6) | 95864.3 (57.3) |  |
| Age (year) |  |  | 0.636 |  |  | 0.099 |
| <50 | 26047 (20.1) | 2138 (4.9) |  | 27875.1 (15.8) | 20727.6 (12.4) |  |
| 50–59 | 46961 (36.3) | 10659 (24.2) |  | 56767.8 (32.2) | 54479.7 (32.6) |  |
| 60–69 | 45636 (35.3) | 24496 (55.7) |  | 71927.0 (40.8) | 72134.7 (43.1) |  |
| ≥70 | 10662 (8.2) | 6683 (15.2) |  | 19534.0 (11.1) | 19923.5 (11.9) |  |
| Index year |  |  | 0.195 |  |  | 0.084 |
| 2006–2009 | 58499 (45.2) | 21607 (49.1) |  | 79064.5 (44.9) | 69248.3 (41.4) |  |
| 2010–2014 | 54277 (42.0) | 19279 (43.8) |  | 76416.3 (43.4) | 74725.8 (44.7) |  |
| 2015–2017 | 16530 (12.8) | 3090 (7.0) |  | 20623.1 (11.7) | 23291.4 (13.9) |  |
| BMI (kg/m^2^) |  |  | 0.419 |  |  | 0.018 |
| <25 | 48824 (37.8) | 9077 (20.6) |  | 57927.7 (32.9) | 53615.4 (32.1) |  |
| 25–30 | 54376 (42.1) | 20281 (46.1) |  | 75316.0 (42.8) | 72175.8 (43.2) |  |
| >30 | 26106 (20.2) | 14618 (33.2) |  | 42860.3 (24.3) | 41474.2 (24.8) |  |
| *APOE* status |  |  | 0.084 |  |  | 0.007 |
| No ε4 allele | 93973 (72.7) | 30353 (69.0) |  | 126409.2 (71.8) | 119752.2 (71.6) |  |
| 1 ε4 Allele | 32557 (25.2) | 12383 (28.2) |  | 45558.3 (25.9) | 43434.1 (26.0) |  |
| 2 ε4 Allele | 2776 (2.1) | 1240 (2.8) |  | 4136.4 (2.3) | 4079.2 (2.4) |  |
| Comorbidity |  |  |  |  |  |  |
| Anxiety | 8698 (6.7) | 3124 (7.1) | 0.015 | 12067.3 (6.9) | 12616.6 (7.5) | 0.027 |
| Coronary artery disease | 859 (0.7) | 3501 (8.0) | 0.365 | 5067.5 (2.9) | 4423.9 (2.6) | 0.014 |
| Depression | 14274 (11.0) | 5273 (12.0) | 0.03 | 20202.8 (11.5) | 21297.3 (12.7) | 0.039 |
| Diabetes mellitus | 1078 (0.8) | 3640 (8.3) | 0.363 | 7335.8 (4.2) | 5026.1 (3.0) | 0.062 |
| Heart failure | 312 (0.2) | 554 (1.3) | 0.118 | 1130.6 (0.6) | 966.8 (0.6) | 0.008 |
| Hypertension | 20537 (15.9) | 20537 (46.7) | 0.705 | 44566.0 (25.3) | 42757.3 (25.6) | 0.006 |
| Kidney disease | 4232 (3.3) | 3118 (7.1) | 0.173 | 8319.4 (4.7) | 7938.0 (4.7) | 0.001 |
| Liver disease | 1202 (0.9) | 740 (1.7) | 0.066 | 2371.3 (1.3) | 2421.0 (1.4) | 0.009 |
| Peripheral vascular disease | 2298 (1.8) | 1375 (3.1) | 0.087 | 4055.9 (2.3) | 4231.1 (2.5) | 0.015 |
| Thyroid disorder | 9013 (7.0) | 3707 (8.4) | 0.055 | 13462.8 (7.6) | 14055.6 (8.4) | 0.028 |
| Medication |  |  |  |  |  |  |
| Benzodiazepines | 29 (0.0) | 67 (0.2) | 0.051 | 106.8 (0.1) | 118.3 (0.1) | 0.001 |
| Blood pressure medications | 20482 (15.8) | 23370 (53.1) | 0.853 | 44731.4 (25.4) | 43370.4 (25.9) | 0.012 |
| Other lipid lowering agents | 38 (0.0) | 101 (0.2) | 0.056 | 292.1 (0.2) | 155.3 (0.1) | 0.020 |
| Tricyclic antidepressants | 13057 (10.1) | 5529 (12.6) | 0.078 | 19592.8 (11.1) | 20863.2 (12.5) | 0.042 |

Values are expressed as number (%). SMD < 0.1 indicates negligible imbalance. Abbreviations: APOE, apolipoprotein E; BMI, body mass index; IPTW: inverse probability of treatment weighting; SMD: standardized mean difference.

Supplementary Table 8. Association between statin use and incident dementia among participants with at least 3 years of observed follow-up.

| Database | Exposure | Case | Control | Person-years | Incidence rate^†^ | Unadjusted HR (95% CI) | Adjusted HR^‡^ (95% CI) |
| --- | --- | --- | --- | --- | --- | --- | --- |
| All of Us | Statin non-user | 1,648 | 71,475 | 459,350.4 | 35.9 | 1 (Ref) | 1 (Ref) |
|  | Statin user | 3,043 | 60,024 | 363,282.2 | 83.8 | 2.36 (2.14–2.61)*** | 2.27 (2.04–2.54)*** |
| UK Biobank | Statin non-user | 568 | 155,266 | 1,221,726.0 | 4.7 | 1 (Ref) | 1 (Ref) |
|  | Statin user | 818 | 120,345 | 840,963.4 | 9.7 | 2.48 (2.04–2.93)*** | 2.72 (2.13–3.40)*** |

Data are derived from weighted proportions and are not absolute numbers.

Abbreviations: CI, confidence interval; HR, hazard ratio; IPTW, inverse probability of treatment weighting.

^†^ Incidence rate per 10,000 person-years.

^‡^ Adjusted for sex, age group, index year, BMI category, *APOE* ε4 status, comorbidities, and concomitant medications. The IPTW from the primary analyses was retained.

*P<0.05, **P<0.01, ***P<0.001.

Supplementary Table 9. Overlap-weighted sensitivity analyses of the association between statin use and incident dementia

| Database | Exposure | Dementia events | ESS | Weighted person-years | Weighted incidence rate^†^ | OW HR (95% CI) | OW-adjusted HR^‡^ (95% CI) |
| --- | --- | --- | --- | --- | --- | --- | --- |
| All of Us | Statin non-user | 1,091 | 37,974.3 | 129,430.0 | 31.3 | 1 (Ref) | 1 (Ref) |
|  | Statin user | 1,829 | 25,033.1 | 111,780.8 | 63.6 | 2.12 (1.94–2.31)*** | 2.18 (2.00–2.38)*** |
| UK Biobank | Statin non-user | 365 | 68,005.3 | 167,709.3 | 7.4 | 1 (Ref) | 1 (Ref) |
|  | Statin user | 401 | 35,067.8 | 135,512.9 | 13.7 | 2.20 (1.85–2.62)*** | 2.75 (2.30–3.29)*** |

Abbreviations: CI, confidence interval; ESS, effective sample size; HR, hazard ratio; OW, overlap weighted.

^†^ Incidence rate per 10,000 overlap-weighted person-years.

^‡^ The overlap-weighted adjusted models additionally included sex, age group, index year, BMI category, APOE ε4 status, comorbidities, and concomitant medications. Robust standard errors were used.

*P<0.05, **P<0.01, ***P<0.001.

Supplementary Table 10. Baseline characteristics of statin users and non-users before and after inverse probability of treatment weighting in the mediation analysis cohort.

|  | Before IPTW | | | After IPTW | | |
| --- | --- | --- | --- | --- | --- | --- |
| Characteristics | Statin non-user (n=32445) | Statin user (n=6108) | SMD | Statin non-user (n=39803.0) | Statin user (n=34223.5) | SMD |
| Sex at birth |  |  | 0.377 |  |  | 0.082 |
| Male | 13682 (42.2) | 3706 (60.7) |  | 18484.6 (46.4) | 17295.1 (50.5) |  |
| Female | 18763 (57.8) | 2402 (39.3) |  | 21318.4 (53.6) | 16928.4 (49.5) |  |
| Age (year) |  |  | 0.725 |  |  | 0.096 |
| <50 | 7119 (21.9) | 254 (4.2) |  | 7372.9 (18.5) | 5268.8 (15.4) |  |
| 50-59 | 10754 (33.1) | 1225 (20.1) |  | 12032.1 (30.2) | 10010.8 (29.3) |  |
| ≥60 | 14572 (44.9) | 4629 (75.8) |  | 20398.0 (51.2) | 18943.8 (55.4) |  |
| BMI (kg/m^2^) |  |  | 0.472 |  |  | 0.040 |
| <25 | 11739 (36.2) | 1062 (17.4) |  | 12876.1 (32.3) | 10457.0 (30.6) |  |
| 25-30 | 13749 (42.4) | 2837 (46.4) |  | 16897.7 (42.5) | 15031.7 (43.9) |  |
| >30 | 6957 (21.4) | 2209 (36.2) |  | 10029.2 (25.2) | 8734.8 (25.5) |  |
| *APOE* status |  |  | 0.117 |  |  | 0.020 |
| No ε4 allele | 23237 (71.6) | 4086 (66.9) |  | 28248.1 (71.0) | 24112.0 (70.5) |  |
| 1 ε4 Allele | 8344 (25.7) | 1767 (28.9) |  | 10423.3 (26.2) | 9029.1 (26.4) |  |
| 2 ε4 Allele | 864 (2.7) | 255 (4.2) |  | 1131.6 (2.8) | 1082.4 (3.2) |  |
| Alcohol use | 30326 (93.5) | 5651 (92.5) |  | 37021.1 (93.0) | 31684.4 (92.6) | 0.017 |
| Smoking status† | 14400 (44.4) | 3440 (56.3) |  | 18570.9 (46.7) | 16349.4 (47.8) | 0.022 |
| Comorbidity |  |  |  |  |  |  |
| Anxiety | 1353 (4.2) | 261 (4.3) | 0.005 | 1720.2 (4.3) | 1605.2 (4.7) | 0.018 |
| Coronary artery disease | 260 (0.8) | 980 (16.0) | 0.571 | 1642.9 (4.1) | 1265.9 (3.7) | 0.022 |
| Depression | 2874 (8.9) | 556 (9.1) | 0.009 | 3661.3 (9.2) | 3528.4 (10.3) | 0.037 |
| Diabetes mellitus | 435 (1.3) | 1157 (18.9) | 0.610 | 2498.3 (6.3) | 1750.1 (5.1) | 0.050 |
| Heart failure | 96 (0.3) | 160 (2.6) | 0.195 | 365.0 (0.9) | 315.7 (0.9) | 0.001 |
| Hypertension | 6410 (19.8) | 3782 (61.9) | 0.950 | 11558.6 (29.0) | 10624.2 (31.0) | 0.044 |
| Kidney disease | 898 (2.8) | 434 (7.1) | 0.201 | 1634.0 (4.1) | 1496.6 (4.4) | 0.013 |
| Liver disease | 218 (0.7) | 65 (1.1) | 0.042 | 348.8 (0.9) | 320.6 (0.9) | 0.006 |
| Peripheral vascular disease | 426 (1.3) | 222 (3.6) | 0.15 | 726.7 (1.8) | 630.8 (1.8) | 0.001 |
| Thyroid disorder | 2153 (6.6) | 557 (9.1) | 0.092 | 2860.0 (7.2) | 2589.0 (7.6) | 0.015 |
| Medication |  |  |  |  |  |  |
| Benzodiazepines | 211 (0.7) | 65 (1.1) | 0.045 | 300.3 (0.8) | 298.9 (0.9) | 0.013 |
| Blood pressure medications | 1817 (5.6) | 2299 (37.6) | 0.845 | 5164.1 (13.0) | 4427.8 (12.9) | 0.001 |
| Other lipid lowering agents | 1361 (4.2) | 450 (7.4) | 0.136 | 2108.7 (5.3) | 2133.4 (6.2) | 0.040 |
| Tricyclic antidepressants | 667 (2.1) | 208 (3.4) | 0.083 | 979.0 (2.5) | 814.3 (2.4) | 0.005 |

Values are expressed as number (%). SMD < 0.1 indicates negligible imbalance. Abbreviations: APOE, apolipoprotein E; BMI, body mass index; IPTW: inverse probability of treatment weighting; SMD: standardized mean difference.

† Smoking status was defined as either current or former smoking.

Supplementary Table 11. Distribution of COQ7 and LDL-C levels in statin users and non-users.

| Biomarker | Group | Mean ± SD | *P*-value† |
| --- | --- | --- | --- |
| COQ7 | Statin non-user (n=39803) | 0.04 ± 0.99 | <0.001 |
|  | Statin user (n=34223.5) | −0.07 ± 1.01 |  |
| LDL-C‡ | Statin non-user (n=37,732) | 0.14 ± 0.92 | <0.001 |
|  | Statin user (n=32,433) | −0.68 ± 0.87 |  |

Data are derived from weighted proportions.

† *P*-values were calculated using linear regression models. ‡ Excluded 1,966 participants with unknown LDL-C.

Abbreviations: COQ7, coenzyme Q7; LDL-C, low-density lipoprotein cholesterol; SD, standard deviation.

Supplementary Table 12. Adjusted differences in baseline COQ7 levels between users of individual statins and non-users in the UKB-PPP cohort.

| Statin type | No. of participants | Adjusted mean difference | P value | Holm-adjusted P value |
| --- | --- | --- | --- | --- |
| Non-user | 32,445 | Reference | — | — |
| Atorvastatin | 1,290 | −0.176 (−0.267 to −0.086) | <0.001 | <0.001 |
| Fluvastatin | 18 | 0.080 (−0.254 to 0.413) | 0.641 | 1 |
| Pravastatin | 176 | −0.081 (−0.375 to 0.212) | 0.587 | 1 |
| Rosuvastatin | 240 | 0.109 (−0.098 to 0.317) | 0.302 | 0.905 |
| Simvastatin | 4,384 | −0.103 (−0.162 to −0.043) | <0.001 | 0.003 |

Linear regression models used the same weights and covariates main analysis. Confidence intervals were calculated using robust sandwich standard errors and were not adjusted for multiple comparisons. Holm correction was applied across the five comparisons with non-users. Differences do not represent within-person changes following treatment initiation.

Abbreviations: CI, confidence interval.

Supplementary Table 13. Pairwise comparisons of baseline COQ7 levels among statin users in the UKB-PPP cohort.

| Comparison | Adjusted mean difference (95% CI) | P value | Holm-adjusted P value |
| --- | --- | --- | --- |
| Atorvastatin vs fluvastatin | −0.276 (−0.631 to 0.079) | 0.127 | 1 |
| Atorvastatin vs pravastatin | −0.077 (−0.398 to 0.244) | 0.638 | 1 |
| Atorvastatin vs rosuvastatin | −0.295 (−0.518 to −0.072) | 0.01 | 0.095 |
| Atorvastatin vs simvastatin | −0.070 (−0.173 to 0.032) | 0.177 | 1 |
| Fluvastatin vs pravastatin | 0.199 (−0.262 to 0.660) | 0.398 | 1 |
| Fluvastatin vs rosuvastatin | −0.019 (−0.419 to 0.381) | 0.926 | 1 |
| Fluvastatin vs simvastatin | 0.206 (−0.141 to 0.552) | 0.244 | 1 |
| Pravastatin vs rosuvastatin | −0.218 (−0.587 to 0.151) | 0.247 | 1 |
| Pravastatin vs simvastatin | 0.007 (−0.310 to 0.323) | 0.967 | 1 |
| Rosuvastatin vs simvastatin | 0.225 (0.013 to 0.436) | 0.037 | 0.334 |

Comparisons were estimated from a separate linear regression model restricted to statin users, using the same weights and covariates main analysis. Differences represent the adjusted mean COQ7 level in the first statin group minus that in the second group. Confidence intervals were calculated using robust sandwich standard errors and were not adjusted for multiple comparisons. Holm correction was applied across the ten pairwise comparisons.

Abbreviations: CI, confidence interval.

Supplementary Table 14. Mediation analyses of the association between statin use and dementia risk with COQ7 and LDL-C.

| Mediators | Path A  β_1_ (95% CI) | Path B  β_2_ (95% CI) | Total effect  β (95% CI) | Direct effect  β (95% CI) | Indirect effect  β (95% CI) | Proportion mediated (%) | Mediated effect  P-value |
| --- | --- | --- | --- | --- | --- | --- | --- |
| COQ7 | −0.10  (−0.12 to −0.09)*** | −0.10  (−0.18 to −0.03)** | 0.0061  (0.0008 to 0.0116)* | 0.0056  (0.0004 to 0.0112)* | 0.0005  (0.0001 to 0.0010)* | 8.2 | 0.032 |
| LDL-C† | −0.83  (−0.85 to −0.81)*** | −0.11  (−0.19 to −0.03)** | 0.0059  (0.0005 to 0.0117)* | 0.0050  (−0.0022 to 0.0123) | 0.0009  (−0.0018 to 0.0040) | 15.3 | 0.588 |

Data are derived from weighted proportions. Abbreviations: β, regression coefficient; CI, confidence interval; COQ7, Coenzyme Q7; LDL-C: low-density lipoprotein cholesterol.

† Excluded 1,966 participants with unknown LDL-C.

**P* <0.05, ***P* <0.01, ****P* <0.001

Supplementary Figure 1. Stratified analysis of the association between statin use and dementia risk in the discovery cohort. Adjusted hazard ratios (HRs) and incidence rates (IRs) for each subgroup are shown. Adjusted HRs are estimated using Cox regression models, adjusting for sex, age group, index year, BMI category, APOE genotype, comorbidities, and medication use, except for the stratification variable in each respective subgroup. IRs are presented per 10,000 person-years.

Supplementary Figure 2. Associations of COQ7 and LDL-C levels with incident dementia in the UKB-PPP cohort. Restricted cubic spline curves from logistic regression models including statin use and (A) COQ7 or (B) LDL-C. Biomarker levels were rank-inverse normal transformed. Three knots were placed at the 10th, 50th, and 90th percentiles, and the median was used as the reference. Solid lines represent odds ratios, and shaded areas indicate pointwise 95% confidence intervals calculated using sandwich standard errors. Curves are displayed between the 5th and 95th percentiles. Horizontal dashed lines indicate an odds ratio of 1, and vertical dotted lines indicate the reference level. P values for overall association and nonlinearity were obtained using robust Wald tests.
